# Interaction Between ITM2B and GLUT9 Links Urate Transport to Neurodegenerative Disorders

**DOI:** 10.3389/fphys.2019.01323

**Published:** 2019-10-22

**Authors:** Asim K. Mandal, David B. Mount

**Affiliations:** ^1^Renal Divisions, Brigham and Women’s Hospital, Harvard Medical School, Boston, MA, United States; ^2^VA Boston Healthcare System, Boston, MA, United States

**Keywords:** integral membrane protein 2B (ITM2B), *SLC2A9* gene, urate transporter GLUT9, neurodegenerative disorders, Danish dementia, retinal dystrophy, *N*-glycosylation

## Abstract

Hyperuricemia plays a critical causative role in gout. In contrast, hyperuricemia has a protective effect in neurodegenerative disorders, including Alzheimer’s Disease. Genetic variation in the *SLC2A9* gene, encoding the urate transporter GLUT9, exerts the largest single-gene effect on serum uric acid (SUA). We report here the identification of two GLUT9-interacting proteins, integral membrane protein 2B (ITM2B) and transmembrane protein 85 (TMEM85), isolated from a human kidney cDNA library using the dual-membrane yeast two-hybrid system. ITM2B is a ubiquitously expressed, *N*-glycosylated transmembrane regulatory protein, involved in familial dementias and retinal dystrophy; the function of TMEM85 is less defined. Using coimmunoprecipitation, we confirmed the physical interaction between ITM2B or TMEM85 and N-terminal GLUT9 isoforms (GLUT9a and GLUT9b) in transfected HEK 293T cells and *Xenopus* oocytes, wherein ITM2B but not TMEM85 inhibited GLUT9-mediated urate uptake. Additionally, co-expression of ITM2B with GLUT9 in oocytes inhibited *N*-glycosylation of GLUT9a more than GLUT9b and stimulated urate efflux by both isoforms. However, urate uptake by *N*-glycosylation and N-terminal deletion GLUT9 mutants was efficiently inhibited by ITM2B, indicating that neither *N*-glycosylation nor the N terminus is necessary for functional interaction of GLUT9 with ITM2B. Notably, ITM2B variants linked to familial Danish dementia and retinal dystrophy significantly attenuated the inhibition of GLUT9–mediated urate influx. We propose ITM2B as a potential regulatory link between urate homeostasis and neurodegenerative disorders.

## Introduction

Elevated serum uric acid (SUA) is a causative risk factor for gout (Choi et al., 2005) and reportedly increases the risk and progression of cardiovascular disease, hypertension, diabetic kidney disease, and chronic kidney disease (Johnson et al., 2018). In contrast, reduced SUA levels increase and elevated SUA levels reduce the risk of neurodegenerative diseases, specifically Alzheimer’s disease (AD) (Ye et al., 2016), Parkinson’s disease (PD) (Schwarzschild et al., 2008; Ascherio et al., 2009), and multiple sclerosis (MS) (Liu et al., 2012).

The circulating SUA in humans is determined by the net balance between production in liver, reabsorption and secretion in renal proximal tubules, and secretion by the intestine (Mandal and Mount, 2015). Within the kidney, filtered urate is reabsorbed in the renal proximal tubule through the coordinated activity of several solute transporters. URAT1, encoded by the *SLC22A12* gene, is the dominant apical urate/anion exchanger in human renal proximal tubule epithelia, reabsorbing urate in exchange with intracellular monocarboxylate anions such as nicotinate (Mandal et al., 2017). The apical OAT10 exchanger (organic anion transporter 10, encoded by the *SLC22A13* gene) also functions in urate-nicotinate exchange in human renal proximal tubule cells, with lesser urate transport activity than URAT1 when expressed in *Xenopus* oocytes (Mandal et al., 2017). The intracellular concentration of monocarboxylate anions that exchange with urate via URAT1 and OAT10 is maintained by the apical Na^+^-dependent monocarboxylate transporters SMCT1 and SMCT2 (Mandal and Mount, 2015).

GLUT9 (glucose transporter 9, encoded by the *SLC2A9* gene) is an electrogenic, high capacity urate uniporter (Anzai et al., 2008; Caulfield et al., 2008; Vitart et al., 2008; Mandal et al., 2017) that mediates the basolateral exit of the reabsorbed urate from proximal tubule cells to the peri-tubular interstitium and the bloodstream. GLUT9 has two isoforms, GLUT9a and GLUT9b, differing in their amino-terminal cytoplasmic domains (Augustin et al., 2004). GLUT9a and GLUT9b transport urate with almost identical affinity (Mandal et al., 2017). However, the two isoforms differ in membrane trafficking; GLUT9a traffics to the basolateral membrane of epithelia whereas GLUT9b is localized at the apical membrane (Kimura et al., 2014). GLUT9a is expressed in human kidney, brain, liver, placenta, lung and leukocytes whereas GLUT9b expression is detected primarily in kidney and placenta (Augustin et al., 2004). Multiple genome-wide association studies (GWAS) have linked variation in SUA to more than forty genes, including *SLC2A9* and several other urate transporter genes (Kottgen et al., 2013; Mandal and Mount, 2015). Notably, variation in *SLC2A9* exert the greatest single-gene effect on SUA. However, very little is known about the regulation of GLUT9.

In this study we report the identification of two GLUT9-interacting proteins, ITM2B (integral membrane protein 2B, also known as BRI2) and TMEM85 (transmembrane protein 85). The human TMEM85 protein is poorly characterized, but appears to have anti-apoptotic activity (Ring et al., 2008). Human ITM2B is a ubiquitously expressed transmembrane protein, most abundant in the brain, placenta, kidney, pancreas, and liver (Pittois et al., 1998).

Mutations in ITM2B cause Familial British Dementia (FBD), Familial Danish Dementia (FDD) (Vidal et al., 1999, 2000), and familial autosomal dominant retinal dystrophy (FRD) (Audo et al., 2014). FBD is caused by a mutation in the normal stop codon (TGA→AGA), generating a C-terminally elongated protein with an extra 11 residues (Vidal et al., 1999). In FDD, a 10-nucleotide duplication (TTTAATTTGT) just three nucleotide before the stop codon also generates an extended ORF with two non-conservative substitutions followed by a distinct C-terminal 11 amino acid extension (Vidal et al., 2000).

FBD and FDD share many similar neuropathological features with AD, and ITM2B also plays a direct role in the pathogenesis of AD. In particular, ITM2B is an inhibitor of APP (amyloid precursor protein) proteolysis and in the absence or dysfunction of ITM2B, production of Aβ (amyloid β) from APP is increased (Tamayev et al., 2012).

We report herein an unexpected function for ITM2B, regulation of urate transport. The physical interaction of ITM2B with GLUT9 isoforms causes inhibition of urate influx and stimulation of urate efflux; in contrast, TMEM85 had no effect on GLUT9 function. The ITM2B mutants associated with FDD and FRD significantly attenuate ITM2B inhibition of urate influx mediated by GLUT9. We propose ITM2B as a novel regulator of SUA and/or cell-specific intracellular urate concentration, and a potential molecular link between uric acid homeostasis and neurodegenerative disorders.

## Materials and Methods

### Animals, Cell Lines and Reagents

The split-ubiquitin dual membrane yeast two-hybrid (MYTH) system and human kidney cDNA libraries were purchased from Dualsystems Biotech (Zurich, Switzerland). Mature female *Xenopus laevis* frogs were purchased from NASCO (Fort Atkinson, WI, United States). The human kidney proximal tubule epithelial cell line PTC-05 (Orosz et al., 2004) was provided by Dr. Ulrich Hopfer. HEK 293T and other cell lines were obtained from ATCC (Manassas, VA, United States). Cell growth media components were purchased from Invitrogen (Carlsbad, CA, United States). Type IV collagen, transferrin, dexamethasone, interferon-gamma, ascorbic acid, sodium selenite (Na_2_SeO_3_), and triiodothyronine (T3) and tricane (Ethyl 3-aminobenzoatemethanesulfonate) were purchased from SIGMA (St. Louis, MO, United States). Affinity-purified rabbit polyclonal anti-GLUT9 antibody was purchased from MBL(Medical & Biological Laboratories Co., Ltd., catalog # BMP027). Rabbit anti-ITM2B antibody (product # HPA029292) and mouse anti-ITM2B antibody (product # SAB1401473) were purchased from SIGMA (St. Louis, MO, United States). Rabbit anti-GAPDH antibody, rabbit anti-Myc antibody, Alexa Fluor 488 conjugated anti-Myc mouse antibody (catalog # A-11029), Alexa Fluor 594 conjugated anti-rabbit IgG (catalog # A-11012) and Sepharose (R) bead conjugated anti-mouse IgG antibody, F(ab’)2 fragment (catalog # 3400) were purchased from Cell Signaling Technology (Danvers, MA, United States). The [^14^C]-urate (specific activity: 50 mCi/mmol) was purchased from Moravec Inc. (Brea, CA, United States). RNeasy Mini Kit and plasmid isolation kit were purchased from QIAGEN (Hilden, Germany).

### Cell Culture

All cells were routinely maintained in their respective appropriate growth medium in a humidified incubator at 37°C with 5% CO_2_. Human PTC-05 cells were grown as described previously (Orosz et al., 2004) on type IV collagen-coated petri dishes. HEK 293T cells and other cells as indicated were grown in Dulbecco’s modified Eagle’s medium (DMEM) following supplier’s instructions.

### Split-Ubiquitin Dual Membrane Yeast Two-Hybrid (MYTH) Screening

The split-ubiquitin dual membrane yeast two-hybrid (MYTH) screening was performed following supplier’s instructions using *Saccharomyces cerevisiae* NMY51. In this screening, we used LexA-VP16-Cub-GLUT9a as the bait protein and human kidney cDNA library proteins fused at their carboxy-terminal end to the NH_2_-terminal half of mutated ubiquitin (NubG) as the prey protein. The full-length human GLUT9a open-reading frame was cloned into the pBT3-N bait vector at an *Sfi1* restriction site. For library screening, approximately ∼5 × 10^6^ yeast transformants were screened per round. Yeast transformants from selection agar plates were grown overnight at 30°C with constant shaking. The yeast transformants were harvested by centrifugation from their 10 ml of overnight culture (O.D. ∼2.0), washed with 1 ml of sterile water, and then suspended in 250 μl of P1 suspension buffer (QIAGEN plasmid isolation kit, Hilden, Germany). Yeast cells were mixed with fine glass beads and then ground with the help of a hand-motor driven pestle for 1 min on ice. Plasmids were then isolated using a QIAGEN plasmid isolation kit.

### Expression Constructs for Human GLUT9/SLC2A9, ITM2B, and TMEM85

For expression of human GLUT9 isoforms, human ITM2B/ITM2B-myc, or human TMEM85/TMEM85-myc in HEK 293T cells, the complementary DNAs (cDNAs) were cloned into the pcDNA3.1(+)/myc-HisA expression vector. For expression in *Xenopus laevis* oocytes, full-length cDNAs were cloned into pGEMHE plasmid vector wherein the cDNA insert is flanked by the *Xenopus laevis* β-globin 5′-UTR and 3′-UTR (Liman et al., 1992).

### Functional Expression in *Xenopus* Oocytes

Studies using *Xenopus laevis* oocytes have been carried out in accordance with the Guide for the Care and Use of Laboratory Animals as adopted and promulgated by the U.S. National Institutes of Health, and were approved by the Institution’s Animal Care and use Committee. Mature female *Xenopus laevis* frogs were subjected to partial ovariectomy under tricane anesthesia (0.17% for 15–20 min) followed by defolliculation of the oocytes as described previously (Mandal et al., 2017). The various *Xenopus laevis* expression constructs in pGEMHE were linearized by *Not1*, *Nhe1*, or *EcoR1* digestion. The cRNAs were *in vitro* synthesized by using T7 RNA polymerase (mMESSAGE mMACHINE; Ambion, Austin, TX, United States) following the supplier’s protocol, isopropanol-precipitated, washed twice with 70% ethanol, dried, dissolved in sterile nuclease-free water and then stored at −80°C. The yield and integrity of the capped cRNA samples were assessed by spectroscopy (at 260 nm) and 1% agarose-formaldehyde gel electrophoresis respectively. About 18 h after isolation, oocytes were microinjected with 50 nl of sterile water, or 50 nl of a cRNA solution containing 25/12.5 ng of the indicated cRNA using fine-tipped micropipettes by a microinjector (World Precision Instrument Inc., Sarasota, FL, United States).

### RNA Extraction and RT-PCR

Total RNA from human kidney proximal tubule epithelial cells (PTC-05) and other cells as indicated were extracted using spin columns with the RNeasy Mini Kit (QIAGEN, GmbH, Germany) following the manufacturer’s instructions. About 2 μg of DNase-treated total RNA, isolated from cells, were primed with poly-dT and random hexamers and then reverse-transcribed using AMV reverse transcriptase (New England Biolabs, Ipswich, MA, United States). Equal amounts of cDNA were used for PCR amplification.

The following intron-spanning primers were used for PCR amplification:

GLUT9b-5S [sense; exon-1B] 5′-GCAGAGGAAGATTCGA ACTGG-3′
GLUT9a-10S [sense; exon-1A] 5′-AATGCCTTGGCAGAGT CTGG-3′
GLUT9b-20S [sense; exon-2] 5′-AACCTGAAAAGTGAAC CATGAAGC-3′
GLUT9a-1A [antisense; exon-3] 5′-CACCACCGACAGGT TGTAGCCGTAG-3′
ITM2B-1S [sense] 5′-TGACGTTCAACTCCGCTCTGGCC CAGAAGG-3′
ITM2B-1A [antisense] 5′-TAGGACTGAGGCAAATAGGT TCCAGCCTTG-3′
TMEM85-2S [sense] 5′-GAGTGGCCATTACGGCCATGAC GGCCCAGGGGGGCCTGGTGGCTA-3′
TMEM85-2A [antisense] 5′-AGCTTCTAGATTACAAAA GCAGTCCTCCACCACTGAACTC-3′
GAPDH-1S [sense] 5′-CGGAGTCAACGGATTTGGTCGT ATTG-3′
GAPDH-1A [antisense] 5′-GACTGTGGTCATGAGTCCT TCCACGA-3′

### Transfection of HEK 293T Cells

HEK 293T cells were grown in DMEM containing 10% FBS. For transfection, 5 × 10^5^ cells were seeded in 6-well tissue culture plates and then next day transfected with 75 pmol of each expression construct using the FuGene 6-reagent (PROMEGA, Madison, WI, United States, catalog # E2311) following manufacturer’s instructions.

### Site-Directed Mutagenesis

Mutations were introduced by site-directed mutagenesis PCR reaction using the QuickChange II XL site-directed mutagenesis kit (Agilent Technologies, Santa Clara, CA, United States, catalog # 210522-5) following the manufacturer’s instructions. Introduced mutations were verified by sequencing. Primer sequences for introducing mutations into *N*-glycosylation sites of GLUT9 isoforms are shown below. Nucleotides mutated for experiments are shown bold and underlined.

GLUT9a-Gly-2S [sense; N90Q] 5′-ATCAAGGCCTTTTA C**C**A**A**GAGTCATGGGAAAGAAGGC-3′
GLUT9a-Gly-2A [antisense; N90Q] 5′-GCCTTCTTTCCCA TGACTC**T**T**G**GTAAAAGGCCTTGATG-3′
GLUT9b-Gly-2S [sense; N61Q] 5′-ATCAAGGCCT TTTAC**C**A**A**GAGTCATGGGAAAGAAGGC-3′
GLUT9b-Gly-2A[antisense; N61Q] 5′-GCCTTCTTT CCCATGACTC**T**T**G**GTAAAAGGCCTTGATG-3′
ITM2B-N170Q-1S → 5′-TGCTATGTGATCCCTCTG **C**A**A**ACTTCCATTGTTATGCCA-3′
ITM2B-N170Q-1A → 5′-TGGCATAACAATGGAAGT **T**T**G**CAGAGGGATCACATAGCA-3′

The following PCR primers were used for generating ITM2B mutants linked to FBD, FDD of familial retinal dystrophy (FRD).

ITMB-1S [sense] → 5′-CTCGGGATCCGGTACCGAGAG ATCTGCCGCCGCGAT-3′
ITMB-FBD-1A [antisense] → 5′GCTAAGCTTAATT TTCCTCAATAATGTTTTTCTTGA CTGTTC**T**AGAACAAATTAAAGTTTCCCGGCAAATTT GTTTTCAAAATGCCG-3′
ITM2B-FDD-1A [antisense] → 5′-GCTTCTAGATCAAT AATGTTTTTCTTGACTGTTCAAGAACAAATTAAAACA AATTAAAGTTGCCACGGCAAATTTGTTTTCAAAATG CCG-3′
ITMB-FRD-1A [antisense] → 5′-GCTCTAGATTAAGAA CAAATTAAAGTT**G**CCACGGCAAATTTGTTTTCAAAA TGC-3′

### *N*-Glycanase Treatment

Clear protein extracts from transfected cells or oocytes expressing proteins as indicated were treated with *N*-glycanase (PROzyme, Hayward, CA, United States, product code # GKE-5006B) following the supplied protocol.

### Immunofluorescence and Immunohistochemistry

HEK 293T cells, cultured in sterile 4-chamber polystyrene tissue culture-treated chamber slides (glass), were transfected with expression constructs (in pcDNA3.1) using the FuGene 6-reagent as described above. Twenty-four hours post-transfection, the cells were fixed and permeabilized with cold methanol for 5 min at −20°C, and then rinsed once with PBS, blocked with 1X casein blocking solution (Vector Laboratories, Burlingame, CA, United States) for 1 h and then incubated with Alexa Fluor 488 conjugated anti-Myc mouse antibody (for ITM2B-myc) or rabbit anti-GLUT9 antibody for 1h followed by rinsing three times with PBS-T buffer. Cells were then incubated with Alexa Fluor 594 conjugated anti-rabbit IgG (for GLUT9 only) for 1h, rinsed three times with TBST buffer (tris-buffered saline with 0.1% tween-20, pH 8.0) and mounted with Vectashield mounting medium (Vector Laboratories, Inc., Burlingame, CA, United States) containing the fluorescent dye 4′,6-diamidino-2-phenylindole (DAPI) for nuclear staining. The fluorescent signal was detected using a confocal Nikon microscope (D-ECLIPSE C1) at 40X (01.0 mm aperture) lateral magnifications.

For immunohistochemistry, normal human kidney sections were obtained by the Renal Pathology Division at Brigham and Women’s Hospital through an IRB-approved protocol (Dr. Vanesa Bijol and Dr. Astrid Weins). Paraffin sections were dewaxed in Xylene, washed six times in absolute ethanol, incubated in a 1:1 diluted solution of 3% hydrogen peroxide and absolute ethanol and then washed in tap water for 3–5 min. After antigen retrieval was achieved in a pressure cooker in Dako citrate buffer pH 6.0 at 120°C for 30 s, sections were rinsed in TBST and then incubated with ITM2B antibody (1:50 dilution) for 45 min then rinsed with TBST for 10 min. Sections were then exposed to Labeled Polymer-HRP anti-rabbit secondary antibody (DakoCytomation, K4011), washed in TBS, and then incubated with DAKO’s DAB + [3,3′-diaminobenzidine (DAB+) substrate-chromogen which results in a brown-colored precipitate at the antigen site] for 3–5 min, followed by washing in tap water and counterstaining with hematoxylin. Pictures of human kidney section were taken using a Nikon microscope (ECLIPSE 90i) at 20X (0.5 mm aperture) and 40X (1.30 mm aperture) lateral magnifications.

### Western Immunoblotting

Western blotting was performed as described previously (Mandal et al., 2017). About 48 h post-transfection/microinjection, cells were lysed by sonication and oocytes (∼100) were lysed using a Teflon homogenizer in lysis buffer (50 mM Tris-HCl, pH 7.5, 50 mM NaCl, 1 mM EDTA, pH 8, 1% Triton X-100) supplemented with protease inhibitors cocktail (Roche, Indianapolis, IN, United States). Western blotting was performed using appropriate antibody. About 30 μg of total protein of lysates was loaded per lane and fractionated using 8% SDS/PAGE gel electrophoresis and then transferred to polyvinylidene difluoride (PVDF) membrane. All of the Western blotting experiments were performed more than three times (*N* > 3) for confirmation; data shown for each figure are from a single representative experiment. Quantitative analysis of the intensity of protein bands in Western blots was performed using KwikQuant Image Manager software (Kindle Biosciences, Greenwich, CT, United States).

### Co-immunoprecipitation

For co-immunoprecipitation, 100 μl of cell lysate (∼300 μg of total proteins) in lysis buffer (150 mM NaCl, 20 mM Tris-Cl, 1% Triton X-100) of transiently transfected HEK 293T cells or microinjected oocytes expressing indicated proteins was mixed with 350 μl of immunoprecipitation buffer (lysis buffer, 0.1% Triton X-100) and 50 μl of protein-A Sepharose slurry (pre-washed and pre-equilibrated) and then incubated in cold room for 2 h with constant rocking. The cell/oocyte lysate were precleared by centrifugation at 2500 × *g* for 5 min. The precleared lysate was then mixed with 20 ml of Sepharose bead conjugated with mouse anti-Myc antibody (IgG2a) for myc-tagged proteins and incubated at 4°C overnight. For co-immunoprecipitation of endogenous GLUT9 with endogenous ITM2B from Caco-2 cell lysate, the precleared lysate (∼300 μg of total protein) was mixed with mouse anti-ITM2B antibody and 40 μl of Sepharose bead conjugated anti-mouse IgG antibody, F(ab’)2 fragment; data shown for each figure are from a single representative experiment. All of the immunoprecipitation experiments shown were performed more than three times (*N* > 3) for confirmation.

### Urate Uptake and Efflux Assays

Twenty four hours post-transfection, transiently transfected HEK 293T cells expressing indicated proteins were harvested separately in 1.7 μl microfuge tube and washed four times with the uptake medium (141 mM NaCl, 2 mM KCl, 1.8 mM CaCl_2_, 1 mM MgCl_2_, 5 mM HEPES, pH 7.4). Then 4 × 10^6^ live cells were suspended in 1 μl of the uptake medium at room temperature containing [^14^C]-urate. After 60 min of incubation in the uptake medium containing [^14^C]-urate (20 μM) at room temperature (∼25°C) in a horizontal shaker-incubator, cells were separately harvested by centrifugation in 1.7 μl microfuge tube, and washed three times with the ice-cold uptake medium to remove external adhering radioisotope. Cells were then lysed by 400 μl of 10% SDS solution and the lysate was completely transferred to scintillation vial for counting by scintillation counter after addition of 2.5 μl of scintillation fluid (Ecoscint, Fischer Scientific, Pittsburgh, PA, United States).

The [^14^C]-urate uptake and efflux experiments in *Xenopus* oocytes were performed as described previously (Mandal et al., 2017). All uptake experiments included at least 20 oocytes in each experimental group; statistical significance was defined as two-tailed *p* < 0.05, and results were reported as means ± SE. All of the uptake experiments shown were performed more than three times (*N* > 3) for confirmation; data shown for each figure are from a single representative experiment.

### Safety

All experiments conformed to institutional biosecurity and biosafety standards.

### Statistics

Statistical analyses including linear regressions and significance were determined by Student’s *t*-test using SigmaPlot software. Transformation of data and curve fitting were made with SigmaPlot (Systat Software, Bangalore, Karnataka, India). Kinetic parameters for the uptake of urate were estimated from the following equation: v = Vmax × S/(Km + S), where v is the rate of substrate uptake (pmol/h/oocyte), S is the substrate concentration in the medium (μm), Km is the Michaelis–Menten constant (μm), and Vmax is the maximum uptake rate (pmol/h/oocyte). These kinetic parameters were determined from analysis of the Eadie–Hofstee plot.

## Results

### Identification of GLUT9-Interacting Proteins Using the Dual Membrane Yeast Two-Hybrid (MY2H) System

Using split ubiquitin membrane yeast two-hybrid (MY2H) screens we identified two GLUT9a-interacting proteins, ITM2B and TMEM85. For this screen, we used LexA-VP16-Cub-GLUT9a as the bait-fusion protein and the human kidney cDNA library proteins, fused at their carboxy-terminal end, to the NH_2_-terminal half of mutated ubiquitin (NubG), as the prey fusion proteins. Out of more than 20 potential GLUT9a-interacting proteins, we selected ITM2B and TMEM85 for further characterization (Supplementary Figures 1A,B).

### ITM2B and TMEM85 Physically Interact With GLUT9 Isoforms

**FIGURE 1:**
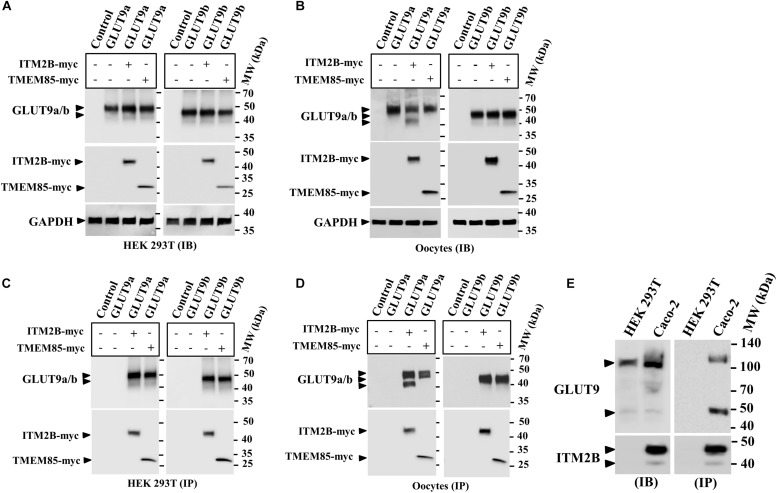
Physical interaction between GLUT9 isoforms and ITM2B-myc or TMEM85-myc: **(A)** Western blot analyses of the lysates of transiently transfected HEK 293T cells, co-expressing GLUT9a/b and ITM2B/TMEM85-myc, using anti-GLUT9 antibody and anti-myc antibody respectively. **(B)** Western blot analyses of the lysates of microinjected oocytes co-expressing GLUT9a/b and ITM2B/TMEM85-myc. Each oocyte was microinjected with 12.5 ng of GLUT9a/b cRNA or a mixture containing 12.5 ng of GLUT9a/b cRNA and 12.5 ng of ITM2B/TMEM-myc cRNA. GAPDH protein band for each sample acts as a loading control. **(C)** Co-immunoprecipitation of GLUT9a/b with ITM2B/TMEM85-myc, using sepharose bead conjugated mouse anti-Myc antibody, from lysates of co-transfected HEK 293T cells co-expressing GLUT9a/b and ITM2B/TMEM85-myc. GLUT9 isoforms were detected by Western blotting using rabbit anti-GLUT9 antibody. **(D)** Co-immunoprecipitation of GLUT9a/b with ITM2B/TMEM85-myc, from lysates of *Xenopus laevis* oocytes co-expressing GLUT9a/b and ITM2B/TMEM85-myc. **(E)** Left panel: Western blot analyses of the lysates (60 μg total protein/lane) of HEK 293T and Caco-2 for endogenous GLUT9 and ITM2B proteins using anti-GLUT9 antibody and anti-ITM2B antibody respectively. Right panel: Endogenous GLUT9 was co-immunoprecipitated with endogenous ITM2B from the lysates of HEK 293T or Caco-2 cells using mouse anti-ITM2B antibody and sepharose (R) bead conjugated anti-mouse IgG antibody, F(ab’)2 fragment. GLUT9 was detected by immunoblotting using rabbit anti-GLUT9 antibody and ITM2B by rabbit anti-ITM2B antibody. IB, immunoblotting; IP, immunoprecipitation.

The results of MY2H screens prompted us to verify the interaction between human ITM2B/TMEM85 and GLUT9a/b in transfected HEK 293T cells and microinjected oocytes co-expressing GLUT9a/b and ITM2B/TMEM85-myc. We first assessed the expression level of GLUT9a/b in the absence and presence of ITM2B/TMEM85-myc. Quantitative analysis (see section Materials and Methods) of the intensity of GLUT9a/b protein band in the Western blots of transfected HEK 293T cell extracts (Figure 1A) or oocyte extracts (Figure 1B) did not show any significant effect of ITM2B/TMEM85-myc expression on GLUT9a/b protein expression levels. We then performed co-immunoprecipitation using Sepharose bead-conjugated mouse anti-myc antibody followed by Western blotting using anti-GLUT9 antibody. The results revealed strong physical interactions between GLUT9 isoforms and ITM2B/TMEM85-myc in both transfected HEK 293T cells (Figure 1C) and *Xenopus* oocytes (Figure 1D).

Notably, co-expression of ITM2B with GLUT9a in oocytes produced two major protein bands for GLUT9a, with molecular sizes ∼50 and ∼45 kDa (Figures 1B,D), without any significant effect on GLUT9b protein (Figures 1B,D). In contrast, GLUT9a/b protein bands remained unaffected when co-expressed with TMEM85 in oocytes (Figures 1B,D). These results suggest that ITM2B might either cause proteolytic cleavage of GLUT9a or might affect post translational modification of GLUT9a in oocytes. However, in transfected HEK 293T cells we did not find such differential effects of ITM2B on the GLUT9 isoforms (Figures 1A,C).

The interactions between over-expressed ITM2B and GLUT9a/b in yeast, transfected cells, or oocytes compelled us to examine physical interactions between endogenous ITM2B and GLUT9. For that, we chose Caco-2 cells, wherein endogenous GLUT9 and ITM2B proteins are easily detectable; HEK 293T cells served as a control, given the lack of endogenous ITM2B protein (Figure 1E, left panel). When we immunoprecipitated ITM2B using mouse anti-ITM2B antibody from Caco-2 and HEK 293T cell lysates and then analyzed the immunoprecipitate by Western blotting using rabbit anti-GLUT9 antibody, we found two protein bands for GLUT9 (∼110 and ∼50 kDa) (Figure 1E, right panel) in the co-immunoprecipitate from Caco-2 cell lysate, but no GLUT9 protein band in the immunoprecipitate from the HEK 293T cell lysate. The results confirm the physical interaction between endogenous ITM2B and GLUT9 proteins.

### ITM2B and GLUT9 Are Co-expressed in Human Renal Proximal Tubule Epithelial Cells

**FIGURE 2:**
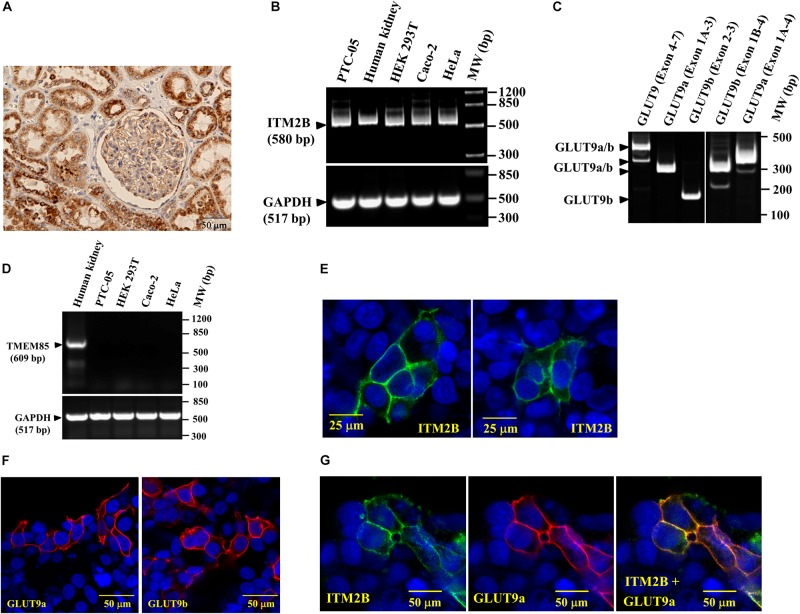
ITM2B and *SLC2A9* gene expression in human renal proximal tubule cells: **(A)** Immunohistochemistry of human kidney section with anti-ITM2B antibody at 20X magnification. ITM2B protein expression (dark brown) is detected in renal proximal tubule epithelial cells. **(B)** RT-PCR detection of ITM2B and GAPDH mRNAs in human kidney, human proximal tubule epithelial cells (PTC-05), HEK 293T, Caco-2, and HeLa cells; GAPDH mRNA expression acts as a control. **(C)** RT-PCR detection of mRNA expression of GLUT9a and GLUT9b in PTC-05 cells. **(D)** RT-PCR detection of TMEM85 mRNA in human kidney with no expression in the cell lines tested here. **(E)** Cell membrane localization of ITM2B-myc protein in transiently transfected HEK 293T cells by confocal immunofluorescence microscopy using Alexa Fluor 488 conjugated mouse anti-myc antibody. **(F)** Cell membrane localization of GLUT9a and GLUT9b proteins in transiently transfected HEK 293T cells by confocal immunofluorescence microscopy using rabbit anti-GLUT9 antibody and Alexa Fluor 594 conjugated anti-rabbit IgG, F(ab’) fragment. **(G)** Cell membrane localization of GLUT9a and ITM2B-myc proteins in transiently transfected HEK 293T cells co-expressing both proteins by confocal immunofluorescence microscopy: GLUT9 protein (red) was detected using rabbit anti-GLUT9 antibody and Alexa Fluor 594 conjugated anti-rabbit IgG and ITM2B-myc (green) was detected using Alexa Fluor 488 conjugated mouse anti-myc antibody. The rightmost panel shows the merged images of ITM2B-myc and GLUT9a protein localization.

Immunohistochemistry of human kidney sections with anti-ITM2B antibody reveals that ITM2B is highly expressed in renal proximal tubules (Figure 2A), wherein GLUT9 expression has previously been reported (Augustin et al., 2004); no signal was seen without ITM2B primary antibody (data not shown). The results of RT-PCR with intron-spanning primers revealed ITM2B mRNA expression in a human proximal tubule epithelial cell line (PTC-05) (Orosz et al., 2004) as well as in many other cell types (Figure 2B), with mRNAs of both isoforms of GLUT9 (GLUT9a and GLUT9b) in PTC-05 cells (Figure 2C); RT-negative control samples were all negative. The RT-PCR products of ITM2B and GLUT9 were confirmed by cloning and sequencing. We could not detect TMEM85 mRNA in PTC-05 cells (Figure 2D); however TMEM85 mRNA was detectable in human kidney, also confirmed by sequencing (Figure 2D).

Confocal immunofluorescence microscopy using anti-Myc antibody in transfected and untransfected (for control) HEK 293T cells reveals that the Myc-tagged ITM2B protein is located predominantly at the cell membrane (Figure 2E), as are the GLUT9 isoforms detected by anti-GLUT9 antibody (Figure 2F). We also detected GLUT9a protein predominantly at the plasma cell membrane when co-expressed with ITM2B (Figure 2G) in co-transfected HEK 293T cells. In untransfected cells, there is no signal for ITM2B and GLUT9 (see Figures 2E,G).

### ITM2B Inhibits Urate Influx and Stimulates Urate Efflux Mediated by GLUT9 Isoforms

**FIGURE 3:**
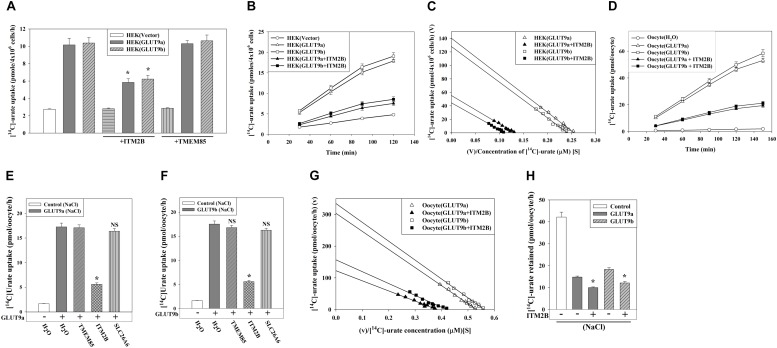
ITM2B inhibits [^14^C]-urate uptake and stimulates [^14^C]-urate efflux mediated by human GLUT9 isoforms. **(A)** The [^14^C]-urate uptake activity of GLUT9a/b expressed alone or co-expressed with ITM2B/TMEM85 in transiently transfected HEK 293T cells (4 × 10^6^) was measured in an isotonic uptake medium containing 20 μM [^14^C]-urate for 1 h at ∼25°C. **(B)** The time course plot of [^14^C]-urate uptake mediated by GLUT9a/b expressed alone or co-expressed with ITM2B in transiently transfected HEK 293T cells (4 × 10^6^). **(C)** ITM2B inhibits [^14^C]-urate uptake mediated by GLUT9a/b following a non-competitive inhibition (mixed) model. The Eadie-Hofstee plot of [^14^C]-urate uptake mediated by GLUT9a/b expressed alone or co-expressed with ITM2B in transiently transfected HEK 293T cells (4 × 10^6^). V, the [^14^C]-urate uptake rate in pmol/4 × 10^6^ cells/h; V/S, [^14^C]-urate uptake rate per concentration (mM) of [^14^C]-urate (S). **(D)** The time course plot of [^14^C]-urate uptake mediated by GLUT9a/b expressed alone or co-expressed with ITM2B in *Xenopus laevis* oocytes measured in an isotonic uptake medium containing 40 μM [^14^C]-urate for 1h at ∼25°C. **(E)** The [^14^C]-urate uptake activity of GLUT9a expressed alone or co-expressed with ITM2B, TMEM85 or a transmembrane protein (SLC26A6) in oocytes was measured in membrane-polarized oocytes (ND96 medium). **(F)** The [^14^C]-urate uptake activity of GLUT9b expressed alone or co-expressed with ITM2B, TMEM85 or SLC26A6 in oocytes was measured in membrane-polarized oocytes. **(G)** ITM2B inhibits [^14^C]-urate uptake mediated by GLUT9a/b following a non-competitive inhibition (mixed) model. The Eadie-Hofstee plot of [^14^C]-urate uptake mediated by GLUT9a/b expressed alone or co-expressed with ITM2B in oocytes. **(H)** ITMB stimulates urate efflux mediated by GLUT9 isoforms. Each oocyte was microinjected with 50 nl of cRNA solution containing 12.5 ng of GLUT9a/b cRNA or a mixture of 12.5 ng of GLUT9a/b cRNA and 12.5 ng of ITM2B/TMEM85/SLC26A6 cRNA. The [^14^C]-urate efflux mediated by GLUT9a/b expressed alone or co-expressed with ITM2B in oocytes was measured in membrane-polarized oocytes for 1 h at ∼25°C. For the [^14^C]-urate efflux experiment, each oocyte was pre-injected with 50 nl of 1500 μM [^14^C]-urate. ^∗^*P* < 0.001 compared with urate efflux in absence of ITM2B.

We subsequently assessed the effect of ITM2B or TMEM85 on the [^14^C]-urate uptake activity of GLUT9a/b in transiently transfected HEK 293T cells. We found that the [^14^C]-urate uptake activity of GLUT9 isoforms was reduced by 54–58% (Figure 3A) in the presence of ITM2B; this [^14^C]-urate uptake was linear over time (Figure 3B). However, the urate transport activity of GLUT9 isoforms was not affected by co-expression with TMEM85 (Figure 3A). The kinetics of GLUT9-mediated urate uptake in transfected HEK293T cells (Supplementary Figure 2) showed that ITM2B caused a decrease in the Vmax of GLUT9a-mediated urate transport from 140.6 to 55.4 pmoles/4 × 10^6^ cells/h and a reduction in apparent Km (Kmapp) from 552 to 414 μM (Figure 3C). Similarly, ITM2B caused the decrease of Vmax of GLUT9b-mediated urate transport from 128 to 45 pmoles/4 × 10^6^ cells/h and the reduction of the apparent Km (Kmapp) from 531 to 395 μM (Figure 3C). These data are consistent with a non-competitive inhibition (mixed) model.

In oocytes, the [^14^C]-urate uptake activity of GLUT9a/b was reduced by 65–70% in presence of ITM2B in membrane-polarized (in ND96 medium, pH 7.4) (Figures 3D,E) or membrane-depolarized oocytes (in Na^+^-free isotonic medium containing 98 mM KCl, pH 7.4) (Figure 3F and Supplementary Figures 3C,D). We did not find any significant effect of TMEM85, or other control transmembrane proteins [SLC26A6, DTDST (SLC26A2), NADC1 and SMCT1] (Figures 3E,F and Supplementary Figures 3A,B) on the urate uptake activity of GLUT9a/b. In oocytes, co-expression with ITM2B decreased the Vmax of GLUT9a-mediated urate uptake from 304 to 122 pmoles/oocyte/h; the apparent Km (Kmapp) was decreased from 571.6 to 319.7 μM (Figure 3G and Supplementary Figure 4). The Vmax of GLUT9b was decreased from 334 to 156 pmoles/oocyte/h and the apparent Km (Kmapp) was decreased from 596 to 373 μM by co-expressed ITM2B (Figure 3G). Again, these data are consistent with a non-competitive inhibition (mixed) model. Of note, the [^14^C]-urate uptake mediated by GLUT9 isoforms was increased 6-7-fold in membrane-depolarized oocytes, with proportionate inhibition by ITM2B (Supplementary Figures 3C,D).

We subsequently assessed the effect of ITM2B on urate *efflux* activity of GLUT9a/b in oocytes. The results show that GLUT9a/b-mediated urate efflux was significantly stimulated (25–30%) by co-expressed ITM2B (Figure 3H and Supplementary Figure 5); this [^14^C]-urate efflux was linear over time (Supplementary Figures 6A,B). Notably, membrane depolarization had almost no effect on urate efflux mediated by GLUT9 isoforms in absence or presence of ITM2B (Supplementary Figure 5).

### ITM2B and GLUT9 Isoforms Are N-Linked Glycosylated Transmembrane Proteins

**FIGURE 4:**
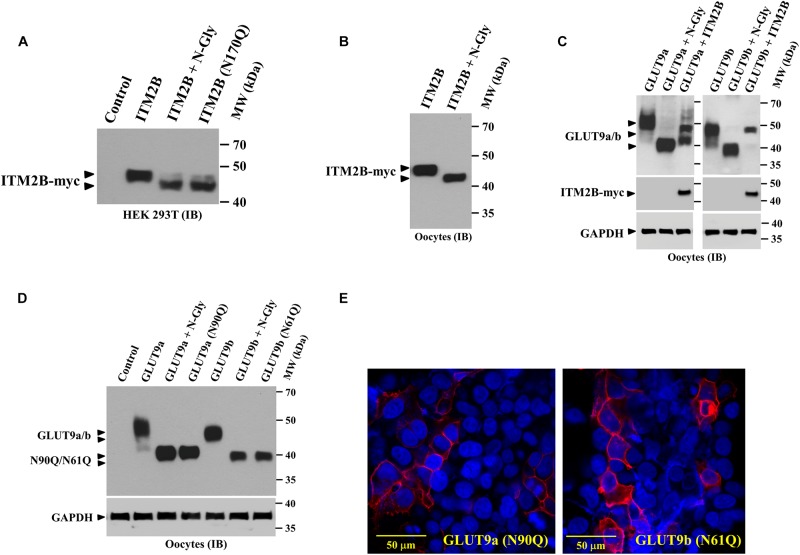
ITM2B and GLUT9 are *N*-glycosylated transmembrane proteins; effect of ITM2B on N-glycosylation of GLUT9 isoforms in oocytes. **(A)** Western blot analysis of the lysate of transfected HEK 293T expressing ITMB-myc protein or *N*-glycosylation-deficient mutant ITM2B (N170Q)-myc protein, digested with and without *N*-glycanase (*N*-gly) *in vitro*. **(B)** Western blot analysis of the lysate of *Xenopus* oocytes expressing ITMB-myc protein, digested with and without *N*-glycanase (*N*-gly) *in vitro*. **(C)** Western blot analysis of the lysate of *Xenopus* oocytes expressing either isoform of GLUT9 (GLUT9a/b) alone or co-expressed with ITM2B-myc, digested with and without *N*-glycanase (*N*-gly) *in vitro*. GAPDH protein band for each sample acts as a loading control. **(D)** Western blot analysis of the lysate of *Xenopus* oocytes expressing either isoform of GLUT9 or their respective N-glycosylation-deficient mutants (GLUT9a-N90Q or GLUT9b-N61Q), generated by site-directed mutagenesis of the shared residues Asn-90 in GLUT9a and Asn-61 in GLUT9b. The molecular sizes of GLUT9 isoforms were compared before and after digestion with *N*-glycanase (*N*-gly). **(E)** Cell membrane localization of *N*-glycosylation-deficient mutants, GLUT9a-N90Q and GLUT9b-N61Q, in transiently transfected HEK 293T cells, was detected by confocal immunofluorescence microscopy using rabbit anti-GLUT9 antibody and Alexa Fluor 594 conjugated anti-rabbit IgG. ITM2B-myc or its mutant and GLUT9 isoforms or its mutants were detected by rabbit anti-myc antibody and rabbit anti-GLUT9 antibody respectively. IB, immunoblotting.

In reducing SDS-PAGE the molecular mass of ITM2B protein appears higher than calculated molecular mass (∼29.2 kDa) (Figure 1E), suggesting that ITM2B could be an *N*-glycosylated protein. After digestion of ITM2B-myc with *N*-glycanase we found a slight reduction (∼ 4 kDa) in the molecular mass of ITM2B-myc (Figures 4A,B) thus confirming ITM2B as an N-linked glycosylated protein. This is in agreement with the previous finding that N-linked glycosylation functions in trafficking of ITM2B to the cell surface (Tsachaki et al., 2011). We then generated *N*-glycosylation mutant of ITM2B by mutating the Asn-170 residue to Gln-170 by site-targeted mutagenesis, and confirmed that ITM2B (N170Q) is the right mutant of ITM2B that lacks N-linked glycosylation (Figure 4A). The digestion of the mutant ITM2B (N170Q) with *N*-glycanase did not reduce the molecular mass (data not shown).

Similarly, we found significant reduction in the molecular mass of GLUT9a and GLUT9b after digestion with *N*-glycanase (Figure 4C) thus confirming that GLUT9 isoforms are N-linked glycosylated proteins. We then generated *N*-glycosylation mutants of GLUT9a/b by mutating the Asn-90 residue of GLUT9a to Gln-90 and Asn-61 residue of GLUT9b to Gln-61 by site-directed mutagenesis, and confirmed by Western blot analysis that GLUT9a (N90Q) and GLUT9b (N61Q) mutants are the dominant N-linked glycosylation sites (Figure 4D). Digestion of GLUT9a(N90Q) and GLUT9b(N61Q) mutants with *N*-glycanase did not reduce the apparent molecular weight of GLUT9a/b (data not shown) thus confirming Asn-90 of GLUT9a and Asn-61 residue of GLUT9b are the only *N*-glycosylation sites.

We next investigated whether N-linked glycosylation of GLUT9 isoforms is required for their cell membrane targeting. Confocal immunofluorescence microscopy using anti-GLUT9 antibody revealed that GLUT9a(N90Q) and GLUT9b(N61Q) mutant proteins are predominantly localized on the plasma membrane (Figure 4E) in transfected HEK 293T cells. Thus, N-linked glycosylation of GLUT9 isoforms is not required for plasma membrane targeting.

### ITM2B Differentially Affects N-Linked Glycosylation of GLUT9 Isoforms in Oocytes

**FIGURE 5:**
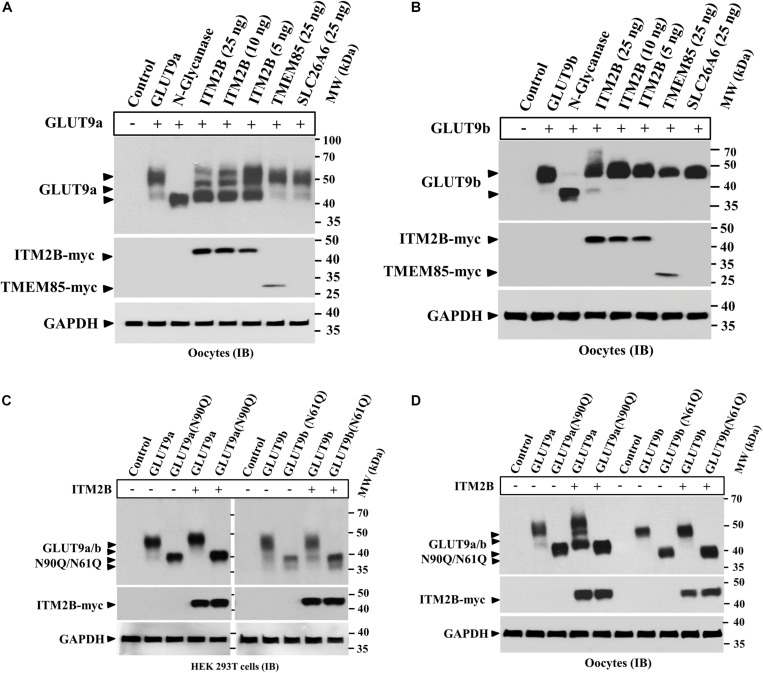
ITM2B differentially affects the N-linked glycosylation level of GLUT9 isoforms in oocytes. **(A)** Western blot analysis of lysates of oocytes expressing GLUT9a alone, or co-expressing GLUT9a and varying amount of ITM2B or fixed amount of other control transmembrane proteins as shown. GAPDH protein band acts as a loading control. For the expression of GLUT9a alone, each oocyte was microinjected with 25 ng of GLUT9a cRNA. For co-expression of GLUT9a with ITM2B, each oocyte was microinjected with a mixture containing 25 ng of GLUT9a cRNA and 5–25 ng of ITM2B cRNA. For co-expression of GLUT9a with TMEM85 or SLC26A6, each oocyte was microinjected with a mixture of 25 ng each of GLUT9a cRNA and TMEM85 or SLC26A6 cRNA. **(B)** Western blot analysis of lysates of oocytes expressing GLUT9b alone, or co-expressing GLUT9b and varying amount of ITM2B or fixed amount of other control transmembrane proteins as shown. GAPDH protein band acts as a loading control. **(C)** Western blot analysis of lysates of transiently transfected HEK 293T cells expressing GLUT9a/b, or their respective *N*-glycosylation-deficient mutants or co-expressing ITM2B-myc with GLUT9a/b, or their respective *N*-glycosylation-deficient mutants. **(D)** Western blot analysis of lysates of oocytes expressing GLUT9a/b, or their respective *N*-glycosylation-deficient mutants or co-expressing ITM2B-myc with GLUT9a/b, or their respective *N*-glycosylation-deficient mutants. GLUT9 isoforms were detected by immunoblotting using rabbit anti-GLUT9 antibody and ITM2B-myc and TMEM85-myc were detected using rabbit anti-myc antibody. IB, immunoblotting.

We found that ITM2B co-expression with GLUT9a in oocytes generated an additional lower molecular size GLUT9a protein band (Figures 1B,D) without significantly affecting GLUT9b. We also found that the lower molecular size GLUT9a protein band appeared in presence of ITM2B approximated the molecular size of GLUT9a digested with *N*-glycanase (Figure 4C). To further demonstrate the differential effect of ITM2B on GLUT9a/b, we microinjected oocytes with a cRNA mixture containing fixed pmol of GLUT9a/GLUT9b cRNA and varying pmol of ITM2B cRNA. We found that a significant fraction of GLUT9a population was deglycosylated in the presence of ITM2B, in a dose-dependent manner (Figure 5A). There was a very modest difference in apparent MW between deglycosylated GLUT9a and the lowest MW band in oocytes co-expressing GLUT9a with ITMB (Figure 4C). Treatment with N-glycanase reduced the MW of this band, consistent with residual core glycoprotein in ITM2B-expressing cells; additionally, the multiple higher MW bands were deglycosylated by *N*-glycanase (data not shown). In contrast to GLUT9a, GLUT9b was not significantly affected by ITM2B co-expression (Figure 5B) indicating differential interaction of ITM2B with GLUT9 isoforms in oocytes.

Next we co-expressed ITM2B with GLUT9a(N90Q) or GLUT9b(N61Q) in HEK 293T cells or oocytes. The results of Western blot analyses show that the molecular mass of GLUT9a(N90Q) or GLUT9b(N61Q) remained unaffected when co-expressed with ITM2B in transfected HEK 293T cells (Figure 5C) or oocytes (Figure 5D), thereby confirming that the protein integrity of GLUT9a/b is unaffected by ITM2B co-expression. Therefore, ITM2B association with GLUT9 isoforms differentially affects *N*-glycosylation level in oocytes, without affecting the integrity and total protein level of GLUT9a/b.

### ITM2B Physically Interacts With and Inhibits the Urate Transport Function of *N*-Glycosylation-Deficient GLUT9 Isoforms

**FIGURE 6:**
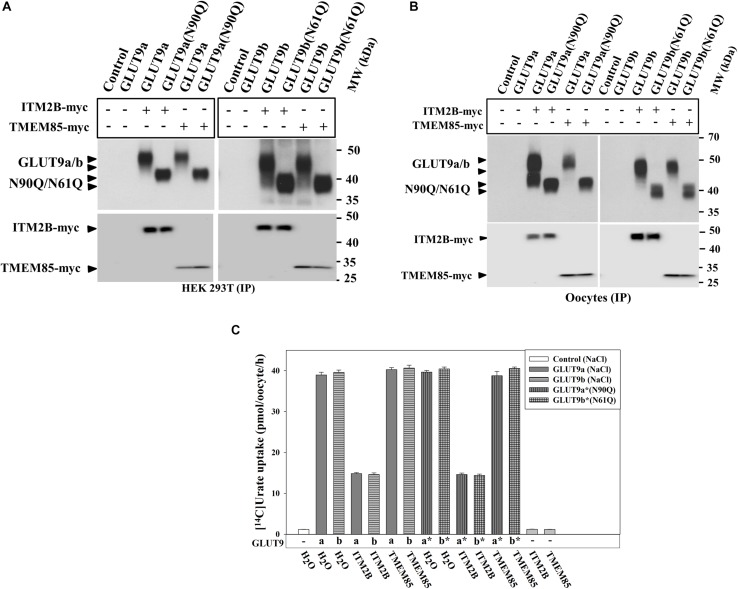
ITM2B physically interacts with N-glycosylation-deficient mutants of GLUT9 isoforms and efficiently inhibits their urate transport function. **(A)** Co-immunoprecipitation of GLUT9a/b or their respective *N*-glycosylation-deficient mutant (GLUT9a-N90Q or GLUT9b-N61Q) with ITM2B/TMEM85-myc, using mouse anti-Myc antibody conjugated with sepharose beads, from lysates of transiently co-transfected HEK 293T cells co-expressing GLUT9a/b or their respective mutants and ITM2B/TMEM85-myc. **(B)** Co-immunoprecipitation of GLUT9a/b or their respective *N*-glycosylation-deficient mutant with ITM2B/TMEM85-myc. The co-immunoprecipitated GLUT9 isoforms or their respective mutants were immunodetected by Western blotting using rabbit anti-GLUT9 antibody and ITM2B/TMEM85-myc was detected using rabbit anti-Myc antibody. **(C)** The [^14^C]-urate uptake activity of GLUT9 isoforms or their respective *N*-glycosylation-deficient mutants, expressed alone or co-expressed with ITM2B/TMEM85, measured in ND96 medium. Each oocyte was microinjected with 50 nl of cRNA solution containing 12.5 ng cRNA of GLUT9a/GLUT9b or the cRNA of their respective mutant or a mixture containing 12.5 ng of GLUT9a/GLUT9b cRNA or the cRNA of their respective mutant and 12.5 ng of cRNA of ITM2B/TMEM85-myc. IP, immunoprecipitation.

N-linked glycosylation is an important determinant of protein structure, function and membrane targeting (Tanaka et al., 2004; Zhou et al., 2005; Waetzig et al., 2010). We sought to determine whether N-linked glycosylation of GLUT9a/b is required for the physical interaction with ITM2B and inhibition of urate transport function by ITM2B. The results of co-immunoprecipitation, followed by Western blotting, clearly revealed that ITM2B or TMEM85 physically interacts with N-glycosylation-deficient mutants of GLUT9 [GLUT9(N90Q) and GLUT9b(N61Q)] in co-transfected HEK 293T cells (Figure 6A) and in *Xenopus* oocytes (Figure 6B). The [^14^C]-urate uptake results also show that GLUT9(N90Q) and GLUT9(N61Q) are functionally equivalent to their wild-type counterparts when assessed in membrane polarized (Figure 6C) or depolarized oocytes (Supplementary Figure 7). We also found that ITM2B very efficiently inhibited (∼65–70% reduction) the urate transport function of GLUT9(N90Q) and GLUT9b(N61Q) (Figure 6C). TMEM85 however, failed to inhibit the [^14^C]-urate transport activity of the *N*-glycosylation deficient mutants (Figure 6C).

### ITM2B Inhibits the Urate Transport Function of N-Terminal Deletion Mutants of GLUT9

**FIGURE 7:**
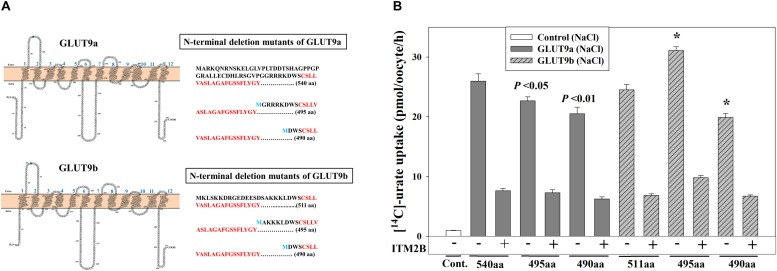
ITM2B inhibits the urate transport activity of N-terminal deletion mutants of GLUT9 isoforms. **(A)** Predicted membrane topology model of the isoforms of GLUT9 (GLUT9a and GLUT9b) with 12 hydrophobic transmembrane domains are shown at the left panels. The *N*-glycosylation site (confirmed by mutational analyses shown in Figure 4D) in the first extracellular loop was highlighted green. The amino acid (aa) sequences of the N-terminal cytoplasmic part of the GLUT9a/b (540/511 aa) and their respective N-terminal deletion mutants (495/490 aa) with starting amino acid M (marked blue) were shown at the right panels with the first transmembrane hydrophobic domains as marked bold red. **(B)** The [^14^C]-urate uptake activity of GLUT9a, GLUT9b, N-terminal deletion mutants of GLUT9a and GLUT9b, expressed alone or co-expressed with ITM2B in *Xenopus* oocytes, was measured in ND96 medium. Each oocyte was microinjected with 50 nl of cRNA solution containing 12.5 ng cRNA of GLUT9a/b or their respective N-terminal deletion mutants. For co-expression of GLUT9 or its mutants with ITM2B, each oocyte was microinjected with 50 nl of cRNA solution containing 12.5 ng of GLUT9a/b or their respective N-terminal deletion mutants and 12.5 ng of ITM2B cRNA. ^∗^*P* < 0.001 compared with the respective urate uptake mediated by GLUT9a(540aa) or GLUT9b(511aa).

The differential effects of ITM2B on the two N-terminal GLUT9a/b variants suggested a potential role for the N-terminal cytoplasmic domain in ITM2B interactions. To assess whether the N-terminal cytoplasmic domain of GLUT9a/b is required for the interaction with ITM2B, we generated N-terminal deletion mutants of GLUT9 isoforms by deleting 45 or 50 amino acids from the N-terminal end of GLUT9a and 16 or 21 amino acids from the N-terminal end of GLUT9b (Figure 7A). The urate uptake results show that the GLUT9a mutant lacking 45 amino acids from its N-terminal end (495 amino acids in total) exhibited an approximately 13% reduction in urate transport activity compared to the normal GLUT9a (540 residues) (Figure 7B). The GLUT9b mutant (495 residues) lacking 16 amino acids from the N-terminal end however showed almost no reduction (rather an ∼20% increase) of urate transport activity compared to the normal GLUT9b (511 residues) (Figure 7B). When the predicted N-terminal cytoplasmic domains were completely deleted for both isoforms, leaving them basically identical in their primary structure, the mutants showed almost equal reduction (∼21%) in their urate transport activity (Figure 7B). A stretch of five amino acid residues (GRRRK in GLUT9a and AKKKL in GLUT9b) in the N-terminal cytoplasmic domain of GLUT9 generates the difference between the urate transport activities of the N-terminal deletion mutants of GLUT9a and GLUT9b. All of the N-terminal deletion mutants of GLUT9 isoforms remained almost equally sensitive to membrane depolarization-driven stimulation of urate uptake (Supplementary Figure 8). Most importantly, ITM2B was still found to efficiently inhibit the urate uptake mediated by all N-terminal deletion mutants of GLUT9 isoforms (Figure 7B and Supplementary Figure 8), indicating that ITM2B physically interacts with domains elsewhere within the GLUT9 protein.

### Disease-Associated and *N*-Glycosylation-Deficient ITM2B Mutants Exhibit Attenuated Inhibition of Urate Uptake by GLUT9 Isoforms

**FIGURE 8:**
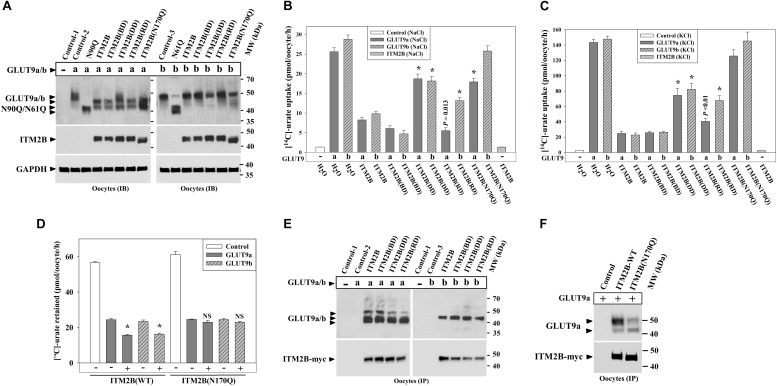
ITM2B mutants associated with familial Danish dementia (FDD), retinal dystrophy (FRD) or *N*-glycosylation (ITM2B-N170Q) exhibit attenuated inhibition of urate uptake by GLUT9 isoforms. **(A)** Western blot analyses of lysates of oocytes expressing GLUT9a/b or co-expressing GLUT9a/b and ITM2B-myc or myc-tagged mutants of ITM2B associated with familial British dementia (FBD), FDD, FRD or *N*-glycosylation (N170Q) in *Xenopus* oocytes. GAPDH protein band acts as a loading control. **(B)** The [^14^C]-urate uptake activity of GLUT9 isoforms expressed alone or co-expressed with ITM2B or its mutants associated with FBD, FDD, FRD or N-glycosylation (N170Q), was measured in ND96 medium. ^∗^*P* < 0.001 compared with urate uptake in presence of normal ITM2B. **(C)** The [^14^C]-urate transport activity of GLUT9 isoforms expressed alone or co-expressed with ITM2B or mutants of ITM2B, was measured in membrane-depolarized oocytes (in Na^+^-free medium containing 98 μM KCl). Note that ITM2B showed proportionate inhibition in both membrane-polarized and depolarized oocytes (Na^+^-free medium containing 98 μM KCl). ^∗^*P* < 0.001 compared with urate uptake mediated by GLUT9 in presence of normal ITM2B. **(D)** ITMB stimulates urate efflux mediated by GLUT9 isoforms but the ITM2B(N170Q) does not. For [^14^C]-urate efflux experiment in *Xenopus laevis* oocytes, each oocyte was pre-injected with 50 nl of 1500 μM [^14^C]-urate and then subjected to urate efflux for 1h in ND96 medium. ^∗^*P* < 0.001 compared with urate efflux mediated by GLUT9 in absence of ITM2B. NS, statistically non-significant. **(E)** Co-immunoprecipitation of GLUT9a/b with myc-tagged ITM2B or its mutants, from lysates of oocytes co-expressing GLUT9a/b and ITM2B-myc or its mutants. Each oocyte was microinjected with 50 nl of cRNA solution containing 12.5 ng of GLUT9a/b cRNA or a mixture of cRNAs containing 12.5 ng of GLUT9a/b cRNA and 12.5 ng of cRNA of ITM2B or its mutants. **(F)** Co-immunoprecipitation of GLUT9a with ITM2B-myc or ITM2B(N170Q)-myc, from lysates of oocytes co-expressing GLUT9a and ITM2B-myc or ITM2B(N170Q)-myc. GLUT9 isoforms were immunodetected by Western blotting using rabbit anti-GLUT9 antibody and ITM2B-myc or myc-tagged ITM2B mutant was immunodetected using rabbit anti-Myc antibody. IB, immunoblotting; IP, immunoprecipitation.

To explore whether ITM2B mutations linked to FBD (Vidal et al., 1999), FDD (Vidal et al., 1999), or FRD (Audo et al., 2014) have any noticeable effect on urate transport mediated by GLUT9 isoforms, we generated the FBD, FDD and FRD mutants of ITM2B, and then co-expressed with GLUT9a/b in oocytes. The results of Western blotting showed that the expression of ITM2B or its mutants have similar differential deglycosylation effects on GLUT9 isoforms (Figure 8A). Surprisingly, the *N*-glycosylation-deficient mutant of ITM2B (N170Q) that reportedly shows reduced trafficking to the cell membrane (Tsachaki et al., 2011) also caused similar deglycosylation of GLUT9a, indicative of a preserved tight association between the two proteins (see section Discussion).

Urate uptake experiments in oocytes revealed significantly attenuated inhibitory effects of the ITM2B-DD, ITM2B-RD and ITM2B(N170Q) mutants (Figures 8B,C) on GLUT9-mediated urate transport. Quantitatively, we detected 72, 80, 28, and 82% inhibition of GLUT9a-mediated urate uptake activity by ITM2B, ITM2B-BD, ITM2B-DD and ITM2B-RD mutants respectively in membrane-polarized oocytes (Figure 8B); in membrane-depolarized oocytes, the inhibition was approximately 84, 83, 48, and 73% (Figure 8C). For GLUT9b, we found 69, 87, 39, and 57% inhibition of urate transport activity by ITM2B, ITM2B-BD, ITM2B-DD, and ITM2B-RD mutants respectively in membrane-polarized oocytes (Figure 8B); in membrane-depolarized oocytes, the inhibition was 86, 83, 45, and 55% (Figure 8C). The ITM2B-DD mutant thus exhibits significant attenuated inhibition of urate uptake mediated by GLUT9a/b (Figures 8B,C). The ITM2B-RD mutant had a more pronounced attenuated inhibitory effect on GLUT9b than GLUT9a.

Finally, we found that the ITM2B *N*-glycosylation mutant (N170Q) had the lowest inhibitory effect on urate uptake (Figures 8B,C) and lowest stimulatory effect of urate efflux (Figure 8D) mediated by GLUT9 isoforms. Quantitatively, we found approximately 31 and 12% inhibition of GLUT9a mediated urate uptake activity by the ITM2B(N170Q) compared to 71 and 84% inhibition by wild-type ITM2B in membrane polarized and membrane depolarized oocytes respectively (Figures 8B,C). For GLUT9b, we found approximately 11 and 1.4% inhibition of urate transport activity by ITM2B(N170Q) compared to 69 and 86% inhibition by wild-type ITM2B in membrane-polarized and membrane-depolarized oocytes, respectively (Figures 8B,C). However, we did not find any significant difference between ITM2B and ITM2B(N170Q) in the stimulation of urate efflux mediated by GLUT9 isoforms (Figure 8D). To investigate whether the attenuated inhibition of GLUT9-mediated urate transport by ITM2B mutants is due to weaker physical interactions, we performed co-immunoprecipitation experiment using protein extracts of oocytes co-expressing either mutant of ITM2B with GLUT9a/b. The results revealed preserved physical interaction of the ITM2B-BD, ITM2B-DD or ITM2B-RD with GLUT9a/b (Figure 8E), however ITM2B(N170Q) showed significantly weaker interaction with GLUT9a/b (Figure 8F).

## Discussion

The *SLC2A9* gene encoding the urate transporter GLUT9 is a major determinant of serum uric acid levels (SUA) (Anzai et al., 2008; Caulfield et al., 2008; Vitart et al., 2008; Mandal et al., 2017). Despite this importance, the regulation of GLUT9 expression and function has been poorly characterized.

Variation in both SUA and the *SLC2A9* gene have several intriguing associations with human cognition and neurodegenerative disease. Reduced SUA has been associated with an increased risk of AD (Ye et al., 2016), Parkinson’s disease (PD) (Schwarzschild et al., 2008; Ascherio et al., 2009), multiple sclerosis (MS) (Liu et al., 2012), schizophrenia (Reddy et al., 2003), poor cognitive function, and dementia (Schretlen et al., 2007; Euser et al., 2009). For *SLC2A9*, an association between the minor alleles of four *SLC2A9* variants with lesser logical memory has been reported (Houlihan et al., 2010). A SNP (rs6834555) located just 5′ of the *SLC2A9* gene has also been associated with psychotic symptoms in AD (Hollingworth et al., 2012). These associations suggest that GLUT9 is an excellent anchor for studies on the influence of uric acid homeostasis on neurological dysfunction.

We have identified two GLUT9 interacting proteins, ITM2B and TMEM85, isolated from a human kidney cDNA library using the dual membrane yeast two-hybrid system (MYTH). ITM2B is a ubiquitously expressed transmembrane protein (Pittois et al., 1998), as is TMEM85. Mutations in the gene encoding ITM2B have been associated with FBD (Vidal et al., 1999), FDD (Vidal et al., 2000), and an autosomal dominant form of retinal dystrophy (Audo et al., 2014). TMEM85 is less well characterized, but suggested to have anti-apoptotic activity (Ring et al., 2008).

We found ITM2B protein expression in proximal tubules of human kidney (Figure 2A) wherein GLUT9a is expressed (Kimura et al., 2014). We also found a moderate expression level of *ITM2B* mRNA and GLUT9a/b (Figures 2B,C) in a human proximal tubular cell line, PTC-05 (Orosz et al., 2004). TMEM85 is expressed in human kidney, but not in proximal tubular cells. We subsequently confirmed strong physical interaction of ITM2B and TMEM85 with GLUT9a/b in both transfected HEK 293T cells (Figure 1C) and microinjected *Xenopus* oocytes (Figure 1D). Our further analyses indicated that unlike TMEM85, ITM2B deglycosylates GLUT9a more than GLUT9b in oocytes (Figures 1B, 4C, 5A,B,D, 8A).

The results of [^14^C]-urate uptake experiments in transfected HEK 293T cells (Figures 3A,B) and in oocytes (Figures 3D–G, 6C and Supplementary Figures 3A–D) demonstrated that unlike TMEM85, ITM2B co-expression significantly reduced urate uptake mediated by GLUT9a/b (55–60% in transfected HEK 293T cells and 65–70% in oocytes). Kinetic analysis revealed a reduction in both Vmax and apparent Km for uric acid uptake mediated by GLUT9a/b in presence of ITM2B (Figures 3C,G). Quantitative analyses of the intensity of protein bands in the Western blot showed that there is no significant reduction in total GLUT9a/b protein level when co-expressed with ITM2B (Figures 1A,B, 5A,B).

N-linked glycosylation has dramatic regulatory effects for ion channels (Chang et al., 2005) and urate transporters (OAT1 and OAT4) (Tanaka et al., 2004; Zhou et al., 2005). We demonstrate here that GLUT9 isoforms are *N*-glycosylated proteins (Figures 4C,D). However, N-linked glycosylation of GLUT9a/b is not an absolute requirement for trafficking to the plasma membrane (Figures 2F, 4E) or for urate transport function (Figure 6C). The co-expression of ITM2B with GLUT9 did not affect plasma membrane trafficking or N-glycosylation of GLUT9a/b in HEK 293T cells (Figures 1A, 2G). In *Xenopus* oocytes, we noticed a differential effect of ITM2B on *N*-glycosylation of GLUT9a versus GLUT9b (Figures 1B,D, 4C, 5A,B,D, 8A) but ITM2B almost equally inhibited the urate transport activities of the functional *N*-glycosylation mutants of GLUT9a and GLUT9b (Figure 6C). Given the differential effects on deglycosylation of the two GLUT9a/b N-terminal variants, we hypothesized that the N-terminal cytoplasmic domain might interact with ITM2B. However, the N-terminal deletion mutants of both GLUT9a and GLUT9b retain urate transport function and remain sensitive to inhibition by ITM2B (Figure 7B), indicating that the GLUT9 N-terminal domains are dispensable for interaction with ITM2B. We hypothesize that the chaperonin-like function of the BRICHOS domain of ITM2B (Willander et al., 2011) confers a tight association of ITM2B with GLUT9, during biosynthesis and beyond, resulting in shared inhibition of both *N*-glycosylation and urate transport. However, the tight co-association of GLUT9 and ITM2B does not appear to modulate trafficking of GLUT9 to the plasma membrane, with no evident intracellular retention of the transporter protein within co-transfected HEK293T cells (Figure 2G). Additionally, the evident activation of GLUT9-mediated efflux by ITM2B (Figure 3H) suggests a functional interaction between the two proteins at the cell membrane.

Notably, we found that ITM2B mutants associated with familial Danish dementia (FDD) (Vidal et al., 2000) and FRD (Audo et al., 2014) showed significantly attenuated inhibition of GLUT9-mediated urate influx (Figures 8B,C). The inhibition by ITM2B and the attenuated inhibition by ITM2B mutants (ITM2B-DD; ITM2B-RD; ITM2B-N170Q) were almost proportionate in both membrane-polarized (Figure 8B) and depolarized (Figure 8C) oocytes. The ITM2B mutant associated with FBD did not, however, show any attenuated inhibition on GLUT9 urate uptake. The *N*-glycosylation mutant of ITM2B (N170Q) showed the most significantly attenuated inhibition (10–30% inhibition by ITM2B-N170Q) of GLUT9-mediated urate uptake with more attenuated inhibition on GLUT9b (2–11% inhibition) than GLUT9a (12–30% inhibition) thus suggesting for inhibition of urate transport function of GLUT9a/b, the physical interaction between ITM2B and GLUT9a/b on the cell membrane might be required. However, we did not find any significant difference between ITM2B and ITM2B(N170Q) in the stimulation of urate efflux mediated by GLUT9 isoforms (Figure 8D) thus indicating regulation of GLUT9-mediated urate efflux might be different from the urate influx effect, which is additionally supported by previous our observation that membrane depolarization had almost no effect on urate efflux mediated by GLUT9 isoforms in the absence or presence of ITM2B (Supplementary Figure 5).

The significantly attenuated inhibition of GLUT9-mediated urate influx by ITM2B-FDD (Figures 8B,C) (Vidal et al., 2000) suggests possible association of GLUT9 function with memory or cognitive function. Several studies have linked uric acid with neuroprotection and cognitive function (Cipriani et al., 2012; Ye et al., 2016) and urate transporters are expressed in human choroid plexus (Alebouyeh et al., 2003). URAT1 and GLUT9 expressed in mouse astrocytes reportedly protect dopaminergic neurons from cellular oxidative stress (Cipriani et al., 2012) through intracellular accumulation of uric acid.

The co-expression of ITM2B with GLUT9 in renal proximal tubular cells suggests that the interaction of these proteins may modulate renal urate excretion and thus circulating SUA levels. However, it remains to be determined whether the attenuated inhibition of GLUT9-mediated urate influx by ITM2B mutants in RD and DD patients affects their SUA. Additionally, queries of genome-wide association databases do not indicate an effect of common ITM2B variants on circulating SUA (data not shown). Alternatively, the ITM2B-GLUT9 interaction might not affect circulating SUA but might affect the compartmentalization of uric acid within the CNS and other organ systems. In this regard, the role of GLUT9 in CNS urate homeostasis is largely unexplored. Future studies of the GLUT9-ITM2B interaction in human neuronal and astrocyte systems will be required to discern how this interaction affects intracellular and extracellular urate homeostasis within the CNS.

The ITM2B point-mutant associated with retinal dystrophy (RD) (Audo et al., 2014) exhibited attenuated inhibitory effects predominantly on GLUT9b-mediated urate transport (Figures 8B,C), suggesting that this form of genetic retinal dystrophy might be associated with altered urate transport. Patients with diabetic macular edema have significantly higher concentration of uric acid in vitreous humor and serum compared to their normal counterpart (Krizova et al., 2011). Rats with inherited retinal degeneration have approximately 150% higher SUA compared to their normal counterparts (Chusova et al., 1982). Significant increases (2–3 fold) in uric acid level in the retina and the optic nerve, after reperfusion of the blood flow by unclipping of the common carotid arteries in rats, have been reported to damage both retina and optic nerve (Sano et al., 1992). Further studies of GLUT9 and ITM2B interactions in the retina might eventually help uncover the pathogenic mechanism(s) of retinal disease associated with ITM2B mutations and help clarify the role of ocular uric acid homeostasis in retinal disease.

## Conclusion

In summary, this study provides compelling evidence that ITM2B is a regulator of GLUT9-mediated urate transport, with potential roles in both systemic and cellular uric acid homeostasis. These findings also identify ITM2B as a molecular link between urate homeostasis and neurodegenerative disorders.

## Data Availability Statement

All datasets generated for this study are included in the manuscript/Supplementary Files.

## Ethics Statement

The animal study was reviewed and approved by Brigham and Women’s Hospital.

## Author Contributions

AM designed and performed the experiments, analyzed the data, and wrote the manuscript. DM conceived the study and finalized the manuscript.

## Conflict of Interest

The authors declare that the research was conducted in the absence of any commercial or financial relationships that could be construed as a potential conflict of interest.

## References

[B1] Alebouyeh M. Takeda M. Onozato M. L. Tojo A. Noshiro R. Hasannejad H. (2003). Expression of human organic anion transporters in the choroid plexus and their interactions with neurotransmitter metabolites. *J. Pharmacol. Sci.* 93 430–436. 10.1254/jphs.93.430 14737013

[B2] Anzai N. Ichida K. Jutabha P. Kimura T. Babu E. Jin C. J. (2008). Plasma urate level is directly regulated by a voltage-driven urate efflux transporter URATv1 (SLC2A9) in humans. *J. Biol. Chem.* 283 26834–26838. 10.1074/jbc.C800156200 18701466

[B3] Ascherio A. LeWitt P. A. Xu K. Eberly S. Watts A. Matson W. R. (2009). Urate as a predictor of the rate of clinical decline in Parkinson disease. *Arch. Neurol.* 66 1460–1468. 10.1001/archneurol.2009.247 19822770 PMC2795011

[B4] Audo I. Bujakowska K. Orhan E. El Shamieh S. Sennlaub F. Guillonneau X. (2014). The familial dementia gene revisited: a missense mutation revealed by whole-exome sequencing identifies ITM2B as a candidate gene underlying a novel autosomal dominant retinal dystrophy in a large family. *Hum. Mol. Genet.* 23 491–501. 10.1093/hmg/ddt439 24026677

[B5] Augustin R. Carayannopoulos M. O. Dowd L. O. Phay J. E. Moley J. F. Moley K. H. (2004). Identification and characterization of human glucose transporter-like protein-9 (GLUT9): alternative splicing alters trafficking. *J. Biol. Chem.* 279 16229–16236. 10.1074/jbc.M312226200M312226200 14739288

[B6] Caulfield M. J. Munroe P. B. O’Neill D. Witkowska K. Charchar F. J. Doblado M. (2008). SLC2A9 is a high-capacity urate transporter in humans. *PLoS Med.* 5:e197. 10.1371/journal.pmed.0050197 18842065 PMC2561076

[B7] Chang Q. Hoefs S. van der Kemp A. W. Topala C. N. Bindels R. J. Hoenderop J. G. (2005). The beta-glucuronidase klotho hydrolyzes and activates the TRPV5 channel. *Science* 310 490–493. 10.1126/science.1114245 16239475

[B8] Choi H. K. Mount D. B. Reginato A. M. (2005). Pathogenesis of gout. *Ann. Intern. Med.* 143 499–516. 16204163 10.7326/0003-4819-143-7-200510040-00009

[B9] Chusova G. G. Ostapenko I. A. Shabanova M. E. Eliseeva R. F. (1982). [Changes in the blood uric acid levels in patients with retinitis pigmentosa and in rats with hereditary retinal degeneration]. *Biull. Eksp. Biol. Med.* 94 21–23. 7150729

[B10] Cipriani S. Desjardins C. A. Burdett T. C. Xu Y. Xu K. Schwarzschild M. A. (2012). Protection of dopaminergic cells by urate requires its accumulation in astrocytes. *J. Neurochem.* 123 172–181. 10.1111/j.1471-4159.2012.07820.x 22671773 PMC3438313

[B11] Euser S. M. Hofman A. Westendorp R. G. Breteler M. M. (2009). Serum uric acid and cognitive function and dementia. *Brain* 132(Pt 2), 377–382. 10.1093/brain/awn316 19036766

[B12] Hollingworth P. Sweet R. Sims R. Harold D. Russo G. Abraham R. (2012). Genome-wide association study of Alzheimer’s disease with psychotic symptoms. *Mol. Psychiatry* 17 1316–1327. 10.1038/mp.2011.125 22005930 PMC3272435

[B13] Houlihan M. Wyatt N. D. Harris S. E. Hayward C. Gow A. J. Marioni R. E. (2010). Variation in the uric acid transporter gene (SLC2A9) and memory performance. *Hum. Mol. Genet.* 19 2321–2330. 10.1093/hmg/ddq097 20197412

[B14] Johnson R. J. Bakris G. L. Borghi C. Chonchol M. B. Feldman D. Lanaspa M. A. (2018). Hyperuricemia, acute and chronic kidney disease, hypertension, and cardiovascular disease: report of a scientific workshop organized by the national kidney foundation. *Am. J. Kidney Dis.* 71 851–865. 10.1053/j.ajkd.2017.12.009 29496260 PMC7286363

[B15] Kimura T. Takahashi M. Yan K. Sakurai H. (2014). Expression of SLC2A9 isoforms in the kidney and their localization in polarized epithelial cells. *PLoS One* 9:e84996. 10.1371/journal.pone.0084996 24409316 PMC3883675

[B16] Kottgen A. Albrecht E. Teumer A. Vitart V. Krumsiek J. Hundertmark C. (2013). Genome-wide association analyses identify 18 new loci associated with serum urate concentrations. *Nat. Genet.* 45 145–154. 10.1038/ng.2500 23263486 PMC3663712

[B17] Krizova L. Kalousova M. Kubena A. Benakova H. Zima T. Kovarik Z. (2011). Increased uric acid and glucose concentrations in vitreous and serum of patients with diabetic macular oedema. *Ophthalmic Res.* 46 73–79. 10.1159/000322994 21242702

[B18] Liman E. R. Tytgat J. Hess P. (1992). Subunit stoichiometry of a mammalian K+ channel determined by construction of multimeric cDNAs. *Neuron* 9 861–871. 10.1016/0896-6273(92)90239-a 1419000

[B19] Liu B. Shen Y. Xiao K. Tang Y. Cen L. Wei J. (2012). Serum uric acid levels in patients with multiple sclerosis: a meta-analysis. *Neurol. Res.* 34 163–171. 10.1179/1743132811Y.0000000074 22333889

[B20] Mandal A. K. Mercado A. Foster A. Zandi-Nejad K. Mount D. B. (2017). Uricosuric targets of tranilast. *Pharmacol. Res. Perspect.* 5:e00291. 10.1002/prp2.291 28357121 PMC5368959

[B21] Mandal K. Mount D. B. (2015). The molecular physiology of uric acid homeostasis. *Annu. Rev. Physiol.* 77 323–345. 10.1146/annurev-physiol-021113-170343 25422986

[B22] Orosz D. E. Woost P. G. Kolb R. J. Finesilver M. B. Jin W. Frisa P. S. (2004). Growth, immortalization, and differentiation potential of normal adult human proximal tubule cells. In Vitro. *Cell Dev. Biol. Anim.* 40 22–34. 14748622 10.1290/1543-706X(2004)40<22:GIADPO>2.0.CO;2

[B23] Pittois K. Deleersnijder W. Merregaert J. (1998). cDNA sequence analysis, chromosomal assignment and expression pattern of the gene coding for integral membrane protein 2 B. *Gene* 217 141–149. 10.1016/s0378-1119(98)00354-0 9795190

[B24] Reddy R. Keshavan M. Yao J. K. (2003). Reduced plasma antioxidants in first-episode patients with schizophrenia. *Schizophr. Res.* 62 205–212. 10.1016/s0920-9964(02)00407-3 12837516

[B25] Ring G. Khoury C. M. Solar A. J. Yang Z. Mandato C. A. Greenwood M. T. (2008). Transmembrane protein 85 from both human (TMEM85) and yeast (YGL231c) inhibit hydrogen peroxide mediated cell death in yeast. *FEBS Lett.* 582 2637–2642. 10.1016/j.febslet.2008.06.042 18586032

[B26] Sano Y. Kanematsu E. H. Yoshiura M. Iwamoto T. Takizawa N. Tokuhisa T. (1992). Uric acid as biochemical marker for retinal and optic nerve damage after occlusion and reperfusion of common carotid and vertebral arteries in rat. *Jpn. J. Ophthalmol.* 36 76–83. 1635299

[B27] Schretlen D. J. Inscore A. B. Jinnah H. A. Rao V. Gordon B. Pearlson G. D. (2007). Serum uric acid and cognitive function in community-dwelling older adults. *Neuropsychology* 21 136–140. 10.1037/0894-4105.21.1.136 17201536

[B28] Schwarzschild M. A. Schwid S. R. Marek K. Watts A. Lang A. E. Oakes D. (2008). Serum urate as a predictor of clinical and radiographic progression in Parkinson disease. *Arch. Neurol.* 65 716–723. 10.1001/archneur.2008.65.6.nct70003 18413464 PMC2574855

[B29] Tamayev R. Matsuda S. Arancio O. D’Adamio L. (2012). beta- but not gamma-secretase proteolysis of APP causes synaptic and memory deficits in a mouse model of dementia. *EMBO Mol. Med.* 4 171–179. 10.1002/emmm.201100195 22170863 PMC3376850

[B30] Tanaka M. Xu W. Zhou F. You G. (2004). Role of glycosylation in the organic anion transporter OAT1. *J. Biol. Chem.* 279 14961–14966. 10.1074/jbc.M400197200 14749323

[B31] Tsachaki M. Serlidaki D. Fetani A. Zarkou V. Rozani I. Ghiso J. (2011). Glycosylation of BRI2 on asparagine 170 is involved in its trafficking to the cell surface but not in its processing by furin or ADAM10. *Glycobiology* 21 1382–1388. 10.1093/glycob/cwr097 21752865 PMC3167477

[B32] Vidal R. Frangione B. Rostagno A. Mead S. Revesz T. Plant G. (1999). A stop-codon mutation in the BRI gene associated with familial British dementia. *Nature* 399 776–781. 10.1038/21637 10391242

[B33] Vidal R. Revesz T. Rostagno A. Kim E. Holton J. L. Bek T. (2000). A decamer duplication in the 3’ region of the BRI gene originates an amyloid peptide that is associated with dementia in a Danish kindred. *Proc. Natl. Acad. Sci. U.S.A.* 97 4920–4925. 10.1073/pnas.080076097 10781099 PMC18333

[B34] Vitart V. Rudan I. Hayward C. Gray N. K. Floyd J. Palmer C. N. (2008). SLC2A9 is a newly identified urate transporter influencing serum urate concentration, urate excretion and gout. *Nat. Genet.* 40 437–442. 10.1038/ng.106 18327257

[B35] Waetzig G. H. Chalaris A. Rosenstiel P. Suthaus J. Holland C. Karl N. (2010). N-linked glycosylation is essential for the stability but not the signaling function of the interleukin-6 signal transducer glycoprotein 130. *J. Biol. Chem.* 285 1781–1789. 10.1074/jbc.M109.075952 19915009 PMC2804336

[B36] Willander H. Hermansson E. Johansson J. Presto J. (2011). BRICHOS domain associated with lung fibrosis, dementia and cancer–a chaperone that prevents amyloid fibril formation? *FEBS J.* 278 3893–3904. 10.1111/j.1742-4658.2011.08209.x 21668643

[B37] Ye B. S. Lee W. W. Ham J. H. Lee J. J. Lee P. H. Sohn Y. H. (2016). Does serum uric acid act as a modulator of cerebrospinal fluid Alzheimer’s disease biomarker related cognitive decline? *Eur. J. Neurol.* 23 948–957. 10.1111/ene.12969 26917248

[B38] Zhou F. Xu W. Hong M. Pan Z. Sinko P. J. Ma J. (2005). The role of N-linked glycosylation in protein folding, membrane targeting, and substrate binding of human organic anion transporter hOAT4. *Mol. Pharmacol.* 67 868–876. 10.1124/mol.104.007583 15576633

