# Supplementary material for: Interaction Between ITM2B and GLUT9 Links Urate Transport to Neurodegenerative Disorders
Source: Front Physiol. 2019 Oct 22;10:1323. doi: 10.3389/fphys.2019.01323 (PMC6818471; doi:10.3389/fphys.2019.01323)
Supplement: Supplementary file 1 [file Data_Sheet_1.PDF]

Supplementary Material:

Supplementary Figure 1

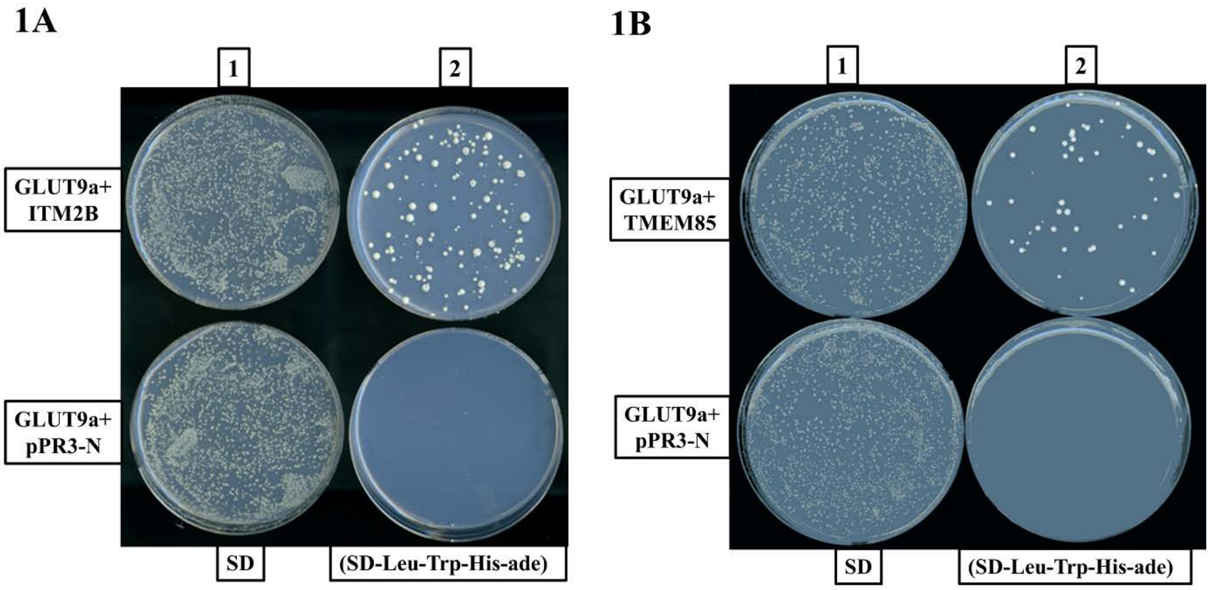

Supplementary Figure 2

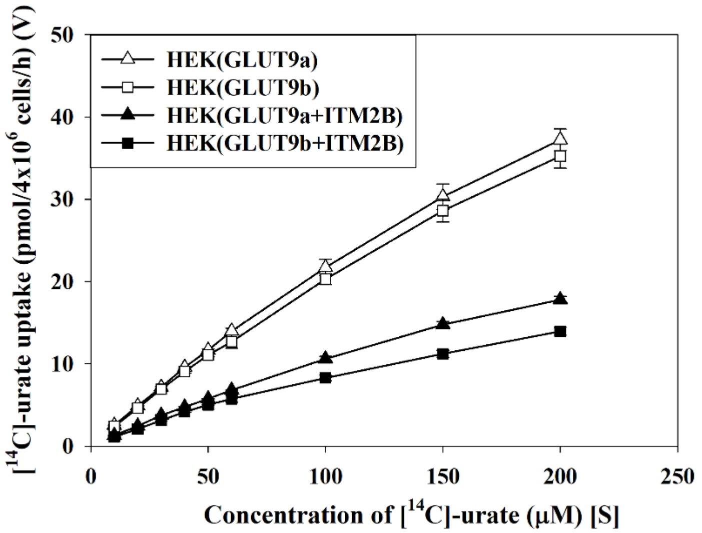

Supplementary Figure 3

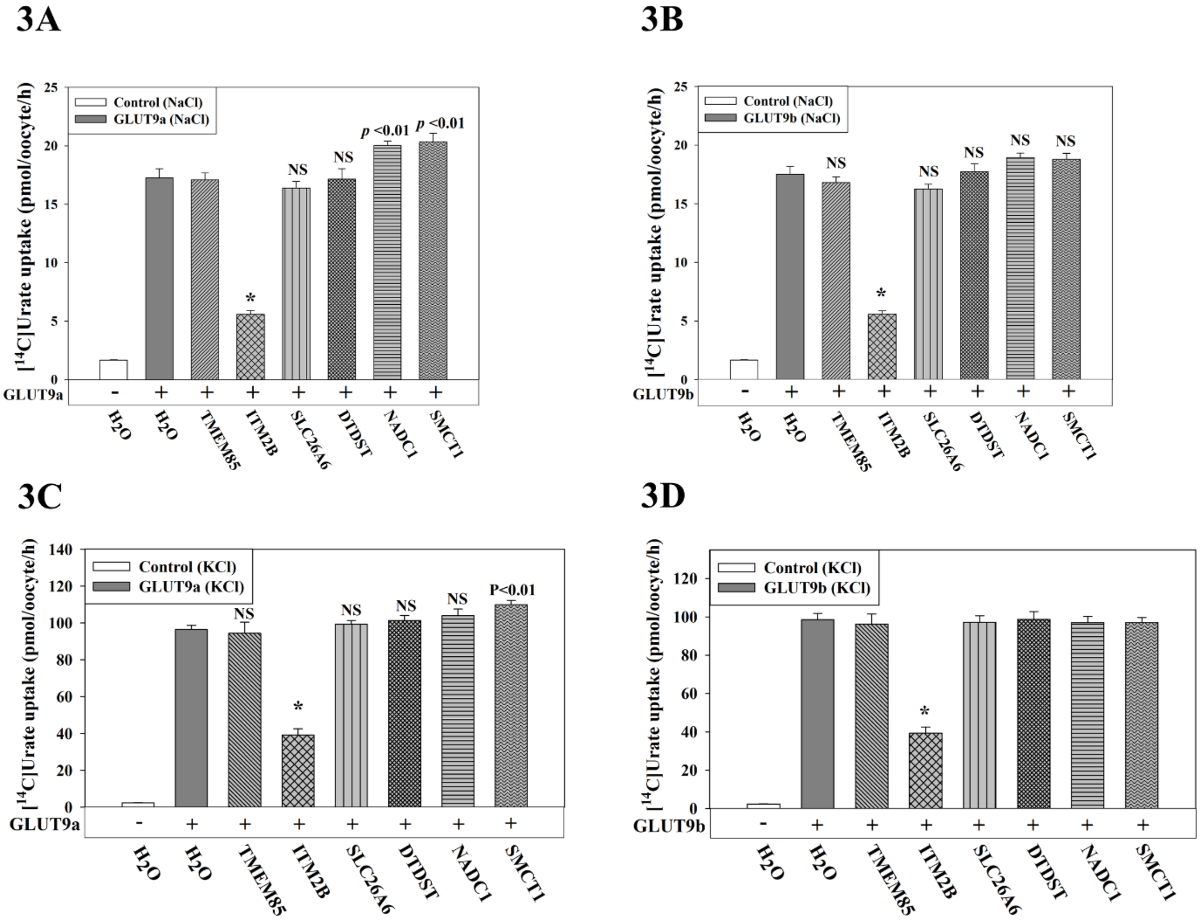

Supplementary Figure 4

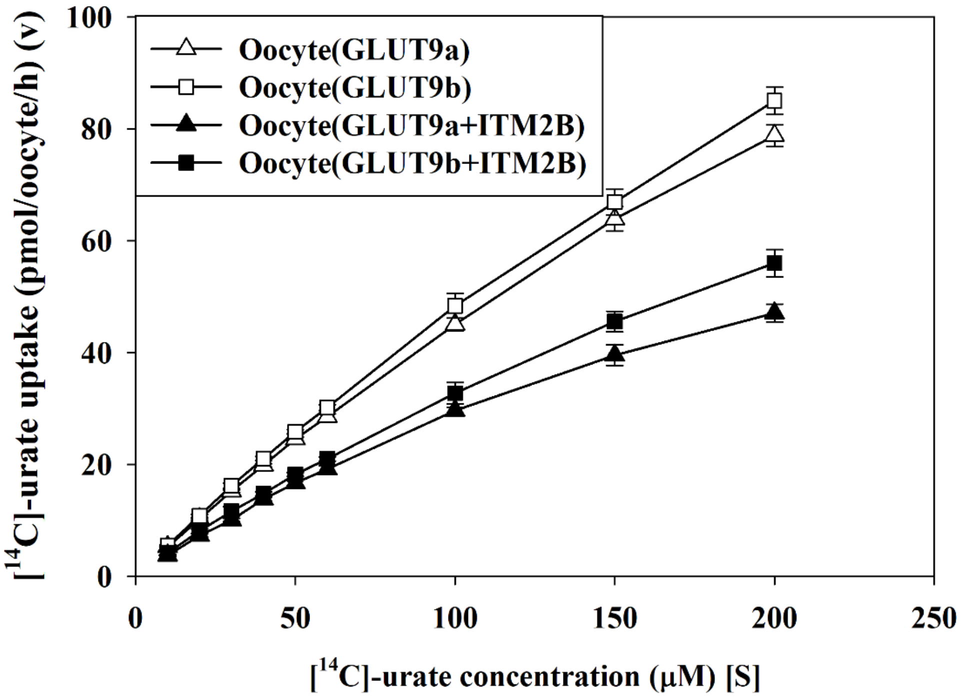

Supplementary Figure 5

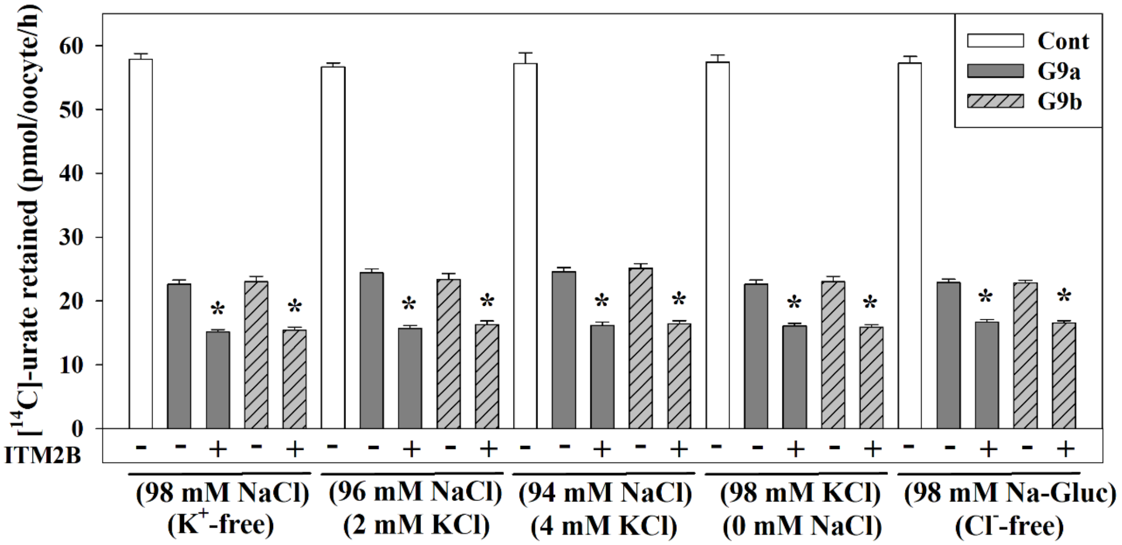

Supplementary Figure 6

6A

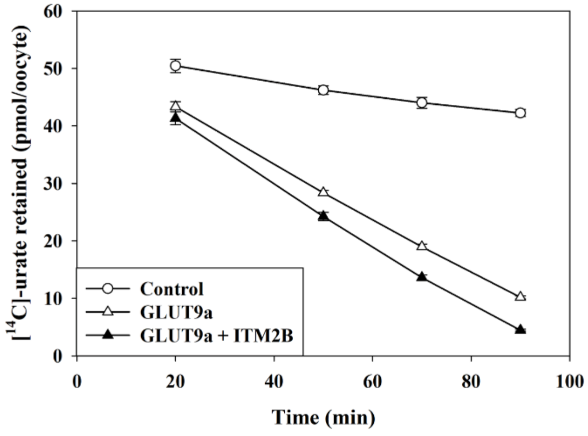

6B

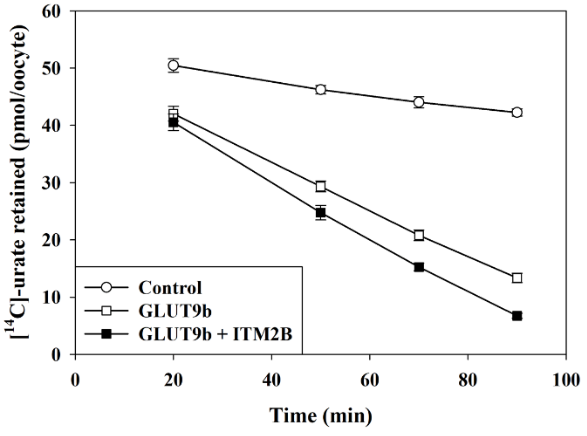

Supplementary Figure 7

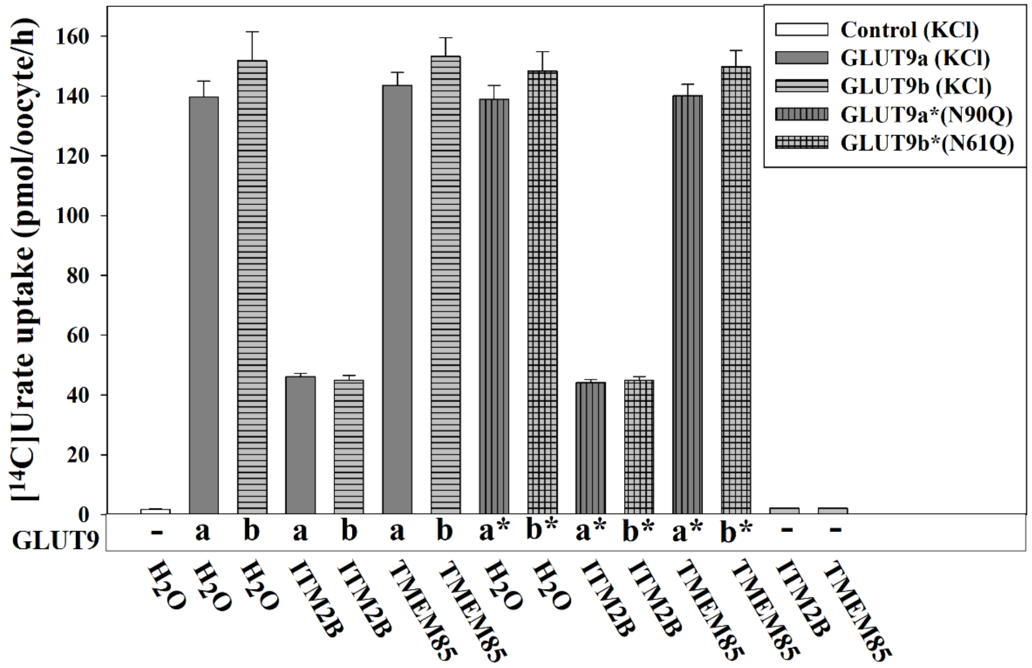

Supplementary Figure 8

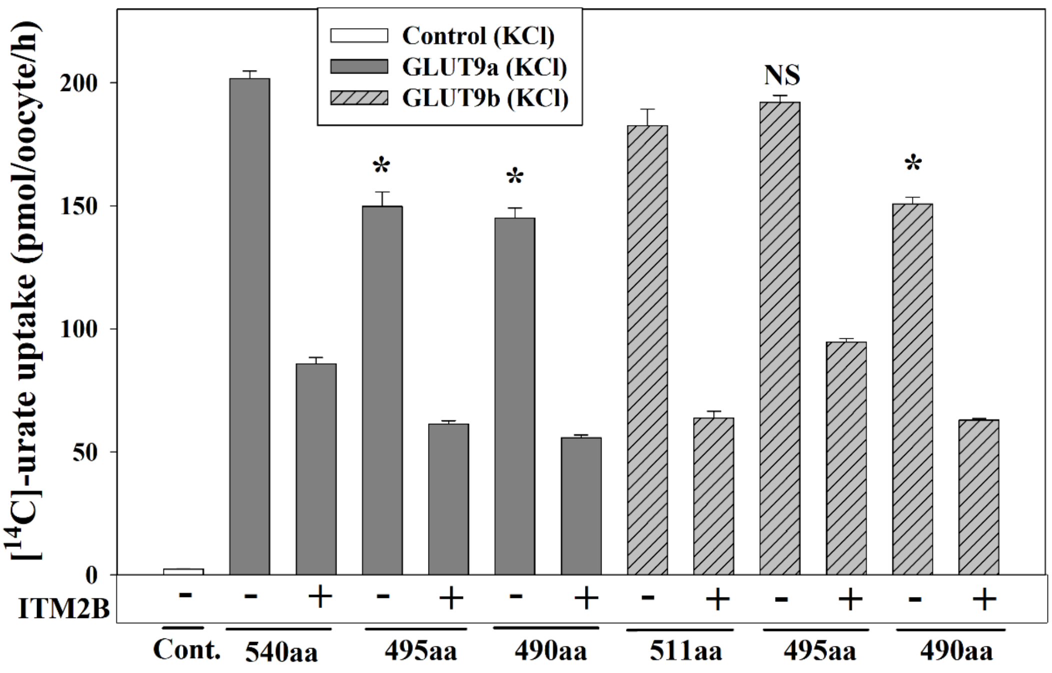

LEGENDS OF SUPPLEMENTARY FIGURES

Supplementary Figure 1

Identification of GLUT9-interacting proteins using the dual-membrane yeast two-hybrid (MY2H) system. (**A**) Identification of ITM2B as a GLUT9a-interacting protein using MY2H system: Panel 1 shows all yeast transformants with bait fusion protein and prey fusion protein (upper part) or no prey fusion protein (lower part; vector only) growing on an SD agar plate. Panel 2 shows that the interaction between LexA-VP16-Cub-GLUT9a bait fusion protein and ITM2B-NubG prey fusion protein allowed yeast transformants to grow into bigger colonies on SD-Leu-Trp-His-ade (Leu, Trp, His, and adenine are omitted) selection agar plate (upper part). In absence of interaction between bait protein and prey fusion protein yeast transformants could not grow on SD-Leu-Trp-His-ade selection agar plate (lower part). (**B**) Identification of TMEM85 as a GLUT9a-interacting protein using MY2H system: Panel 1 shows all yeast transformants with bait fusion protein and prey fusion protein (upper part) or no prey fusion protein (lower part; vector only) growing on an SD agar plate. Panel 2 shows that the interaction between LexA-VP16-Cub-GLUT9a bait and TMEM85-NubG prey allowed yeast transformants to grow into bigger colonies on SD-Leu-Trp-His-ade (Leu, Trp, His, and adenine are omitted) selection agar plate (upper part). In absence of interaction between bait protein and prey fusion protein yeast transformants could not grow on SD-Leu-Trp-His-ade selection agar plate (lower part). pPR3-N is the control vector for prey fusion protein expression. All of the experiments shown were performed more than three times (N>3) for confirmation; data for each figure are from a single representative experiment.

Supplementary Figure 2

The kinetics of inhibition of GLUT9-mediated urate uptake by ITM2B in transiently transfected HEK 293T cells. The kinetic curves of [^{14}C]-urate uptake mediated by GLUT9a/GLUT9b expressed alone or co-expressed with ITM2B in transiently transfected HEK 293T cells ($4x10^6$) measured in an isotonic uptake medium (141 mM NaCl, 2 mM KCl, 1.8 mM $CaCl_2$, 1 mM $MgCl_2$, 5 mM HEPES, pH 7.4) containing increasing concentration of [^{14}C]-urate (10-200 μM) for 1h at room temperature (~25^0C) in the extracellular medium. All of the experiments shown were performed more than three times (N>3) for confirmation; data for each figure are from a single representative experiment.

Supplementary Figure 3

ITM2B inhibits urate influx mediated by GLUT9 isoforms. (**A**) ITM2B inhibits [^{14}C]-urate uptake mediated by human GLUT9a. The [^{14}C]-urate uptake activity of GLUT9a expressed alone or co-expressed with ITM2B, TMEM85 or other transmembrane proteins (SLC26A6, DTDST, NADC1 or SMCT1) in *Xenopus laevis* oocytes was measured in membrane-polarized oocytes (in ND96 medium, pH 7.4). (**B**) ITM2B inhibits [^{14}C]-urate uptake mediated by human GLUT9b. The [^{14}C]-urate uptake activity of GLUT9b expressed alone or co-expressed with ITM2B, TMEM85 or other transmembrane proteins (SLC26A6, DTDST, NADC1 or SMCT1) in *Xenopus laevis* oocytes was measured in membrane-polarized oocytes (in ND96 medium, pH

7.4). (**C**) ITM2B inhibits GLUT9a-mediated [^{14}C]-urate uptake in membrane-depolarized oocytes. The [^{14}C]-urate uptake activity of GLUT9a expressed alone or co-expressed with ITM2B, TMEM85 or other transmembrane proteins (TMEM85, SLC26A6, DTDST, NADC1 or SMCT1) in *Xenopus laevis* oocytes was measured at room temperature (~25°C) for 1h in membrane-depolarized oocytes (in Na^+-free isotonic medium, pH 7.4; achieved by substituting NaCl in ND96 medium by KCl). Note that [^{14}C]-urate uptake mediated by GLUT9a is increased ~6-fold in membrane-depolarized oocytes compared to membrane-polarized oocytes (**Figure 3E**) and ITM2B proportionately inhibited this increased [^{14}C]-urate uptake. (**D**) ITM2B inhibits GLUT9b-mediated [^{14}C]-urate uptake in membrane-depolarized oocytes. The [^{14}C]-urate uptake activity of GLUT9b expressed alone or co-expressed with ITM2B, TMEM85 or other transmembrane proteins (TMEM85, SLC26A6, DTDST, NADC1 or SMCT1) in *Xenopus laevis* oocytes was measured at room temperature (~25°C) for 1h in membrane-depolarized oocytes (in Na^+-free isotonic medium, pH 7.4; achieved by substituting NaCl in ND96 medium by KCl). Each oocyte was microinjected with 50 nl of cRNA solution containing 12.5 ng of GLUT9a cRNA or a mixture of 12.5 ng of GLUT9a cRNA and 12.5 ng of ITM2B, TMEM85, SLC26A6, DTDST, NADC1 or SMCT1 cRNA. Note that [^{14}C]-urate uptake mediated by GLUT9b is increased ~6-fold in membrane-depolarized oocytes compared to membrane-polarized oocytes (**Figure 3F**) and ITM2B proportionately inhibited this increased [^{14}C]-urate uptake. All data are mean $\pm$ s.e.m. with n = 12–15 oocytes per group. All experiments shown were performed more than three times (N>3) for confirmation. Data for each figure are from a single representative experiment. Asterisk, $P < 0.001$ compared with urate efflux in absence of ITM2B. NS, statistically non-significant.

Supplementary Figure 4

The kinetics of inhibition of GLUT9-mediated urate uptake by ITM2B in *Xenopus laevis* oocyte expression system. The kinetic curve of [^{14}C]-urate uptake mediated by GLUT9a/GLUT9b expressed alone or co-expressed with ITM2B in *Xenopus laevis* oocytes measured in an isotonic uptake medium (ND96 medium, pH 7.4) containing increasing concentration of [^{14}C]-urate (10-200 μM) in the extracellular medium. All data are mean $\pm$ s.e.m. with n = 12–15 oocytes per group. All of the experiments shown were performed more than three times (N>3) for confirmation; data for each figure are from a single representative experiment.

Supplementary Figure 5

ITM2B stimulates GLUT9-mediated [^{14}C]-urate efflux in oocytes. The [^{14}C]-urate efflux mediated by GLUT9a/GLUT9b expressed alone or co-expressed with ITM2B in *Xenopus laevis* oocytes was measured after incubation at room temperature (~25°C) for 1h in various isotonic media (pH 7.4) as indicated that affect resting membrane potential of oocytes. For the [^{14}C]-urate efflux experiment, each oocyte was pre-injected with 50 nl of 1500 μM [^{14}C]-urate. Pre-injected oocytes were then incubated in for 30 min at 16°C for recovery, washed and then subjected to efflux for 1h at room temperature (~25°C). G9a, GLUT9a; G9b, GLUT9b; Cont, Control. Note that the [^{14}C]-urate efflux mediated by GLUT9 isoforms remains almost unchanged in all experimental conditions that affects oocyte membrane potential which is in stark contrast with GLUT9-mediated urate uptake (**Figure 3E** and **F**; **Supplemental Figure 3C** and **D**). Asterisk, $P < 0.001$ compared with urate efflux in absence of ITM2B. All data are mean $\pm$ s.e.m. with n =

12–15 oocytes per group. All of the experiments shown were performed more than three times (N>3) for confirmation; data for each figure are from a single representative experiment.

Supplementary Figure 6

The time course plot of urate efflux mediated by GLUT9 isoforms. (**A**) The time course plot of [^{14}C]-urate efflux mediated by GLUT9a expressed alone or co-expressed with ITM2B in *Xenopus laevis* oocytes. (**B**) The time course plot of [^{14}C]-urate efflux mediated by GLUT9b expressed alone or co-expressed with ITM2B in *Xenopus laevis* oocytes. For the [^{14}C]-urate efflux experiment, each oocyte was pre-injected with 50 nl of 1500 μM [^{14}C]-urate. Pre-injected oocytes were then incubated in for 30 min at 16°C for recovery, washed and then subjected to efflux at room temperature (~25°C) for 1h in ND96 medium (pH 7.4; see methods). All data are mean ± s.e.m. with n = 12–15 oocytes per group. All of the experiments shown were performed more than three times (N>3) for confirmation; data for each figure are from a single representative experiment.

Supplementary Figure 7

N-linked glycosylation of GLUT9 isoforms is not essential for urate transport function, voltage sensitivity or inhibition of urate transport by ITM2B. The [^{14}C]-urate uptake activity of GLUT9 isoforms or their respective N-glycosylation-deficient mutants, GLUT9a(N90Q) and GLUT9b(N61Q), expressed alone or co-expressed with ITM2B/TMEM85, was measured at room temperature (~25°C) for 1h in membrane-depolarized oocytes (isotonic Na^{+}-free medium, pH 7.4; achieved by substituting NaCl in ND96 medium by KCl). Each oocyte was microinjected with 50 nl of cRNA solution containing 12.5 ng of GLUT9a/GLUT9b cRNA or cRNA of their respective mutant or, a mixture containing 12.5 ng of GLUT9a/GLUT9b cRNA or cRNA of their respective mutant and 12.5 ng of ITM2B or TMEM85 cRNA. After microinjection, oocytes were incubated in ND96 medium supplemented with pyruvate for 48 hours prior to urate uptake experiments in the indicated medium. Data are mean ± S. E. with n = 12-15 oocytes per group. All of the experiments shown were performed more than three times (N>3) for confirmation; data for each figure are from a single representative experiment.

Supplementary Figure 8

The distinct N-terminal cytoplasmic domains of GLUT9 isoforms are not essential for urate transport function, voltage sensitivity or inhibition of urate transport by ITM2B. The [^{14}C]-urate uptake activity of N-terminal deletion mutants of GLUT9a (45 and 50 amino acids deleted from the N-terminal end) and GLUT9b (16 and 21 amino acids deleted from the N-terminal end), expressed in *Xenopus* oocytes, was measured at room temperature (~25°C) for 1h in membrane-depolarized oocytes (isotonic Na^{+}-free medium pH 7.4; achieved by substituting NaCl in ND96 medium by KCl). Each oocyte was microinjected with 50 nl of cRNA solution containing 25 ng cRNA of GLUT9a/GLUT9b or their respective deletion mutants. After microinjection, oocytes were incubated in ND96 medium, supplemented with pyruvate, for 48 hours prior to urate uptake experiment in the indicated medium. Asterisk, $P < 0.001$ compared with urate uptake mediated

by GLUT9a(540aa) or GLUT9b(511aa). NS, statistically non-significant. Data are mean ± S. E. with n = 12-15 oocytes per group. All of the experiments shown were performed more than three times (N>3) for confirmation; data for each figure are from a single representative experiment.
